# Detection and disease diagnosis trends (2017–2022) for *Streptococcus suis*, *Glaesserella parasuis*, *Mycoplasma hyorhinis*, *Actinobacillus suis* and *Mycoplasma hyosynoviae* at Iowa State University Veterinary Diagnostic Laboratory

**DOI:** 10.1186/s12917-023-03807-w

**Published:** 2023-12-12

**Authors:** Ana Paula Serafini Poeta Silva, Marcelo Almeida, Alyona Michael, Michael C. Rahe, Christopher Siepker, Drew R. Magstadt, Pablo Piñeyro, Bailey L. Arruda, Nubia R. Macedo, Orhan Sahin, Philip C. Gauger, Karen M. Krueger, Robert Mugabi, Jessica S. Streauslin, Giovani Trevisan, Daniel C. L. Linhares, Gustavo S. Silva, Eduardo Fano, Rodger G. Main, Kent J. Schwartz, Eric R. Burrough, Rachel J. Derscheid, Panchan Sitthicharoenchai, Maria J. Clavijo

**Affiliations:** 1 Veterinary Diagnostic and Production Animal Medicine, College of Veterinary Medicine, Iowa State University, Ames, IA USA; 2 United States Department of Agriculture (USDA), Ames, IA USA; 3 Boehringer Ingelheim Animal Health USA Inc, Atlanta, GA USA

**Keywords:** *Streptococcus suis*, *Glaesserella parasuis*, *Mycoplasma hyorhinis*, *Actinobacillus suis*, *Mycoplasma hyosynoviae*, Detection, Diagnosis, Monitoring, Endemic, Swine, Polymicrobial, Disease

## Abstract

**Background:**

Accurate measurement of disease associated with endemic bacterial agents in pig populations is challenging due to their commensal ecology, the lack of disease-specific antemortem diagnostic tests, and the polymicrobial nature of swine diagnostic cases. The main objective of this retrospective study was to estimate temporal patterns of agent detection and disease diagnosis for five endemic bacteria that can cause systemic disease in porcine tissue specimens submitted to the Iowa State University Veterinary Diagnostic Laboratory (ISU VDL) from 2017 to 2022. The study also explored the diagnostic value of specific tissue specimens for disease diagnosis, estimated the frequency of polymicrobial diagnosis, and evaluated the association between phase of pig production and disease diagnosis.

**Results:**

*S. suis* and *G. parasuis* bronchopneumonia increased on average 6 and 4.3%*,* while *S. suis* endocarditis increased by 23% per year, respectively. *M. hyorhinis* and *A. suis* associated serositis increased yearly by 4.2 and 12.8%, respectively. A significant upward trend in *M. hyorhinis* arthritis cases was also observed. In contrast, *M. hyosynoviae* arthritis cases decreased by 33% average/year. Investigation into the diagnostic value of tissues showed that lungs were the most frequently submitted sample, However, the use of lung for systemic disease diagnosis requires caution due to the commensal nature of these agents in the respiratory system, compared to systemic sites that diagnosticians typically target. This study also explored associations between phase of production and specific diseases caused by each agent, showcasing the role of *S. suis* arthritis in suckling pigs, meningitis in early nursery and endocarditis in growing pigs, and the role of *G. parasuis, A. suis, M. hyorhinis* and *M. hyosynoviae* disease mainly in post-weaning phases. Finally, this study highlighted the high frequency of co-detection and -disease diagnosis with other infectious etiologies, such as PRRSV and IAV, demonstrating that to minimize the health impact of these endemic bacterial agents it is imperative to establish effective viral control programs.

**Conclusions:**

Results from this retrospective study demonstrated significant increases in disease diagnosis for *S. suis*, *G. parasuis*, *M. hyorhinis,* and *A. suis*, and a significant decrease in detection and disease diagnosis of *M. hyosynoviae*. High frequencies of interactions between these endemic agents and with viral pathogens was also demonstrated. Consequently, improved control programs are needed to mitigate the adverse effect of these endemic bacterial agents on swine health and wellbeing. This includes improving diagnostic procedures, developing more effective vaccine products, fine-tuning antimicrobial approaches, and managing viral co-infections.

**Supplementary Information:**

The online version contains supplementary material available at 10.1186/s12917-023-03807-w.

## Introduction

*Streptococcus suis* (*S. suis*), *Glaesserella parasuis* (*G. parasuis*), *Mycoplasma hyorhinis* (*M. hyorhinis*), *Actinobacillus suis* (*A. suis*), and *Mycoplasma hyosynoviae* (*M. hyosynoviae*) are among the most significant bacterial pathogens of swine that can cause systemic disease in swine [1]. In addition to their direct impact on pig health and productivity, they are key drivers of antimicrobial use on farms [2]. While these bacteria can be commensals of the upper respiratory tract of pigs, they can also cause severe systemic disease often culminating in increased herd morbidity and mortality [3]. Colonization of piglets by these agents can occur through vertical transmission, contact with the sow during the suckling period, as well as between pigs in the nursery and postweaning stages [4–6].

Although colonization usually occurs early in life and is widespread in the population, various factors such as incubation period, pathogenicity of strains, polymicrobial interactions, commingling of pigs of different ages and sources, and other husbandry and management practices determine disease expression at different phases of the production cycle [3]. Thus, control programs for these agents require a multifaceted approach that enhances immunity and minimizes bacterial load through gilt acclimation (e.g., homologous sow herd exposure), management practices and pig flow, and strategic use of antimicrobials and commercial and autogenous vaccines [7–9].

Disease associated with *S. suis* and *G. parasuis* are commonly observed in suckling and nursery pigs (~ 10 weeks of age) with a spectrum of lesions that vary from arthritis, bronchopneumonia, meningitis, polyserositis, and acute death [10, 11]. *M. hyorhinis* commonly causes polyserositis and arthritis in nursery pigs between 3 and 10 weeks of age [12, 13]. Bronchopneumonia, pleuritis, polyserositis, and acute death are the most common manifestations of *A. suis*-associated disease, which occurs mainly in the grow-finish phase in pigs between 10 and 16 weeks of age, but can also be seen in pre-weaning piglets in high health herds [14–16]. Similarly, the disease associated with *M. hyosynoviae* is commonly observed in later phases of pig development as arthritis in pigs between 12 and 22 weeks of age [17, 18].

Accurate measurement of disease associated with endemic bacterial agents in pig populations is challenging due to their commensal ecology, the lack of disease-specific antemortem diagnostic tests, and the polymicrobial nature of swine diagnostic cases [19]. Therefore, the main objective of this retrospective study was to estimate temporal patterns of agent detection and disease diagnosis for *S. suis*, *G. parasuis*, *M. hyorhinis*, *A. suis*, and *M. hyosynoviae* in porcine tissue specimens submitted to the Iowa State University Veterinary Diagnostic Laboratory (ISU VDL). The study also explored the diagnostic value of specific tissue specimens for disease diagnosis, estimated the frequency of polymicrobial diagnosis and evaluated the association between phase of pig production and disease diagnosis.

## Materials and methods

### Study overview

The ISU VDL is fully accredited by the American Association of Veterinary Laboratory Diagnosticians according to the ISO 17025 since 2007. In 2022, the laboratory received over 88,134 porcine diagnostic cases. A diagnostic case (the unit of analysis) consists of the specimens and associated information provided by the submitting veterinarian for the investigation of a disease event on one swine farm, as well as the test results and diagnostic interpretation provided by the diagnostician. This information is stored in a customized Laboratory Information Management System (LIMS) on a standardized disease diagnosis coding system [20].

The detection of the five bacterial agents in this study was based on standard bacteriological isolation techniques, polymerase chain reaction (PCR) assays, or both. Culture of *S. suis*, *G. parasuis*, and *A. suis* was performed using standard procedures [21], with taxonomic identification by matrix-assisted laser desorption/ionization time of flight mass spectrometry (MALDI-TOF, MALDI Biotyper®, Bruker, Billerica, MA USA) [22]. Due to their fastidious nature, culture of *M. hyorhinis* and *M. hyosynoviae* is not routinely performed. For nucleic acid detection, extraction of *G. parasuis*, *M. hyorhinis*, and *M. hyosynoviae* DNA was performed using the MagMAX™-96 Pathogen RNA/DNA kit (Applied Biosystems™, Carlsbad, CA USA) on the automated Kingfisher™ Flex Purification System (Thermo Fisher Scientific, Inc., Waltham, MA USA) followed by amplification using specific PCRs, as described elsewhere [23, 24]. The detection information is given to the level of sample, and this information is uploaded by laboratory personnel to the ISU VDL LIMS.

For disease diagnosis, a pathological evaluation is conducted by ISU VDL diagnosticians, and diagnostic codes (DxCodes) are assigned to the case based on the interpretation of the clinical history, diagnostic testing, and pathological changes. Derscheid et al. [20] highlights that disease diagnostic codes are a summarization of the case and they then serve as a proxy for querying data for stakeholder support, surveillance, and benchmarking of animal diseases in their herds. Briefly, the DxCodes included in this study were related to body system, insult, lesion, and etiology. A summary of the terms used in this study is given in Supplementary Table 1.

The ISU VDL LIMS database search was performed from January 1, 2017, and December 31, 2022. The search was conducted for diagnostic tests (e.g., culture and/or PCR) associated with the detection of the five agents. Information gathered from the diagnostic test included the type of bacterial agents detected, the specimens and lesions associated with disease associated to these five agents. Concerning disease diagnosis, the DxCode search was conducted based on the list of codes associated with *S. suis*, *G. parasuis*, *A. suis*, *M. hyorhinis,* and *M. hyosynoviae* infection (Supplementary Table 2). After that, analyses of temporal trends in agent detection or disease diagnosis and relevant associations were analyzed at the case level.

### Data management

The data was generated by extracting, integrating, aggregating, and summarizing information within a porcine diagnostic case [25, 26]. A diagnostic case may contain one or more pigs with different agents detected in different specimens or include one or more DxCodes. In this study, a case represented a detection or disease event in a pig population. Three modified datasets of bacteriology, PCR, and DxCode containing aggregated data by accession number were created using functions from *dplyr* and *tidyverse* packages in R (version 4.2.1, R core team 2022).

Overall, bacterial detection datasets included information on pathogen isolated or PCR results (positive, suspect and negative), case year, and specimens used for isolation or PCR testing for each case. With detection datasets, specimens that can support disease diagnosis of the five studied agents were selected and then recoded and grouped into sets. The set of specimens identified as *central nervous system* (CNS) *samples* included brain, brain swab, cerebrospinal fluid, cerebellum, spinal cord, spinal cord swab, and meninges. The *serosal fibrin* set included abdominal fluid, fibrin, fibrin swab, liver surface, lung surface, spleen surface, peritoneal fluid, peritoneal fluid swab, peritoneum, pleura, pleural swab, pleural fluid, heart surface, heart, heart swab epicardium, pericardium, pericardial sac, pericardial fluid, and pericardial swab. The set of *heart valve* includes only heart valvular leaflets. The *joint* set included hoof, joint, joint capsule, joint fluid, joint swab, joint carpus, joint fetlock, synovial fluid, synovial membrane, and synovial tissue. The *kidney* set included kidney and kidney swab. The *liver* set included liver and liver swab. The *lung* set included lung and lung swab. The *spleen* set included spleen. All remaining specimens were classified as *other* or *non-identified*. Thus, cases that lacked identification of specimens (e.g., cases that included “culture”, “assorted” or “tissue” as reported specimens) were also not considered in the analyses of detection trends. To count number of cases with a given specimen each year, a new variable “*agent + specimen*” was created by concatenating the column including pathogen identification and specimen for detection. After that, data were aggregated by year and duplicated accession identifiers were removed, and then counts and trend analyzes were performed using the variable *agent + specimen*, as described in the statistical methods.

The DxCode dataset included variables of animal identification, age, etiology identified, affected body system (cardiovascular, musculoskeletal, nervous, respiratory, and systemic), lesion (arthritis, bronchopneumonia, endocarditis, meningitis, serositis, and sepsis) for each animal in each case. Each case could have included one or a combination of lesions. Research, ancillary and cases that included non-infectious etiology and non-pathological lesions (e.g., anomalies, metabolic, traumas, etc.) were removed. Categorization of case age of pigs into production phase was carried out as follows: 1) *suckling piglets* (0 < x ≤ 3-week-old); 2) *early-nursery* (3 < x ≤ 6-week-old); 3) *late-nursery* (6 < x ≤ 10-week-old); 4) *growing* (10 < x ≤ 16-week-old); and 5) *finishers/adults* (16-week-old < x). Then, a new variable was created, namely “*etiology + lesion*”, where infectious etiology and lesion Dx Codes were concatenated. To count the number of cases for each agent associated with a lesion each year, the matching pair of *etiology + lesion* variable was aggregated by year and duplicated accession identifiers were removed, and then count and trend analyses were performed, as described in the statistical methods.

### Data analyses

#### Trend analyses by agent

The detection rates by year for culture (*S. suis*, *G. parasuis*, and *A. suis*) and PCR (*G. parasuis*, *M. hyorhinis*, and *M. hyosynoviae*) were estimated using binomial regression models. The proportion between number of cases with at least one bacterial isolation or positive PCR result in at least one specimen of interest for each agent divided by total culture or total PCR testing was considered the dependent variable and year the independent variable.

Likewise, disease diagnosis rates per year for each agent by a compatible lesion were calculated using binomial regression models. *S. suis* disease diagnosis trends were estimated for arthritis, bronchopneumonia, endocarditis, meningoencephalitis, sepsis, or serositis cases; *G. parasuis* disease diagnosis trends were estimated for arthritis, bronchopneumonia, meningoencephalitis, sepsis, or serositis; *M. hyorhinis* disease diagnosis trends were estimated for arthritis, bronchopneumonia, or serositis; *A. suis* disease diagnosis trends were estimated for arthritis, bronchopneumonia, sepsis, or serositis; and trends for *M. hyosynoviae* disease were estimated for arthritis. For all these models, the number of disease diagnosis cases for each agent associated with a specific lesion was divided by the total porcine diagnostic cases with a related assigned infectious etiology with the associated lesion by each year (Supplementary Table 3) and was considered the dependent variable, and the year was the independent variable.

#### Relationship between disease diagnosis and specimens of detection

This evaluation aimed to contrast pathogen detection and disease diagnosis by evaluating the detection of target pathogens in specimens of interest and an established disease diagnosis (etiology associated with a lesion). The numerator represented the total number of disease cases with an etiology and associated lesion (*etiology + lesion*), and the denominator included cases that included detection for the given agent along with a specific specimen (*agent + specimen*) but also included a histopathology assessment to guarantee that the case was also evaluated for disease diagnosis. This strategy was chosen due to the absence of specimen information in the pathology dataset and that not all specimens were evaluated for lesions. To illustrate the detection of *S. suis* in CNS samples with *S. suis* meningitis disease, the number of cases of *S. suis* meningitis was divided by the number of cases that simultaneously included *S. suis* detection in CNS samples and DxCodes for any disease.

#### Disease diagnosis by production phase and agent

Comparisons of number of cases by production phase was done using Poisson regressions for each agent within each lesion.

#### Polymicrobial diagnosis

Polymicrobial diagnosis in this study were related to the presence of disease caused by more than one infectious etiology, for example, a DxCode including PRRSV and *S. suis*. The frequencies of polymicrobial diagnosis among the five agents and other porcine pathogens were analyzed using the body systems (cardiovascular, musculoskeletal, nervous, respiratory, and/or systemic). Cases that were given more than two body system DxCodes (e.g., respiratory + cardiovascular; nervous + systemic), i.e., more than one body system was involved in the whole case, were all classified as multisystemic to facilitate interpretation.

## Results

### Bacterial culture frequencies and detection trends

**Fig. 1:**
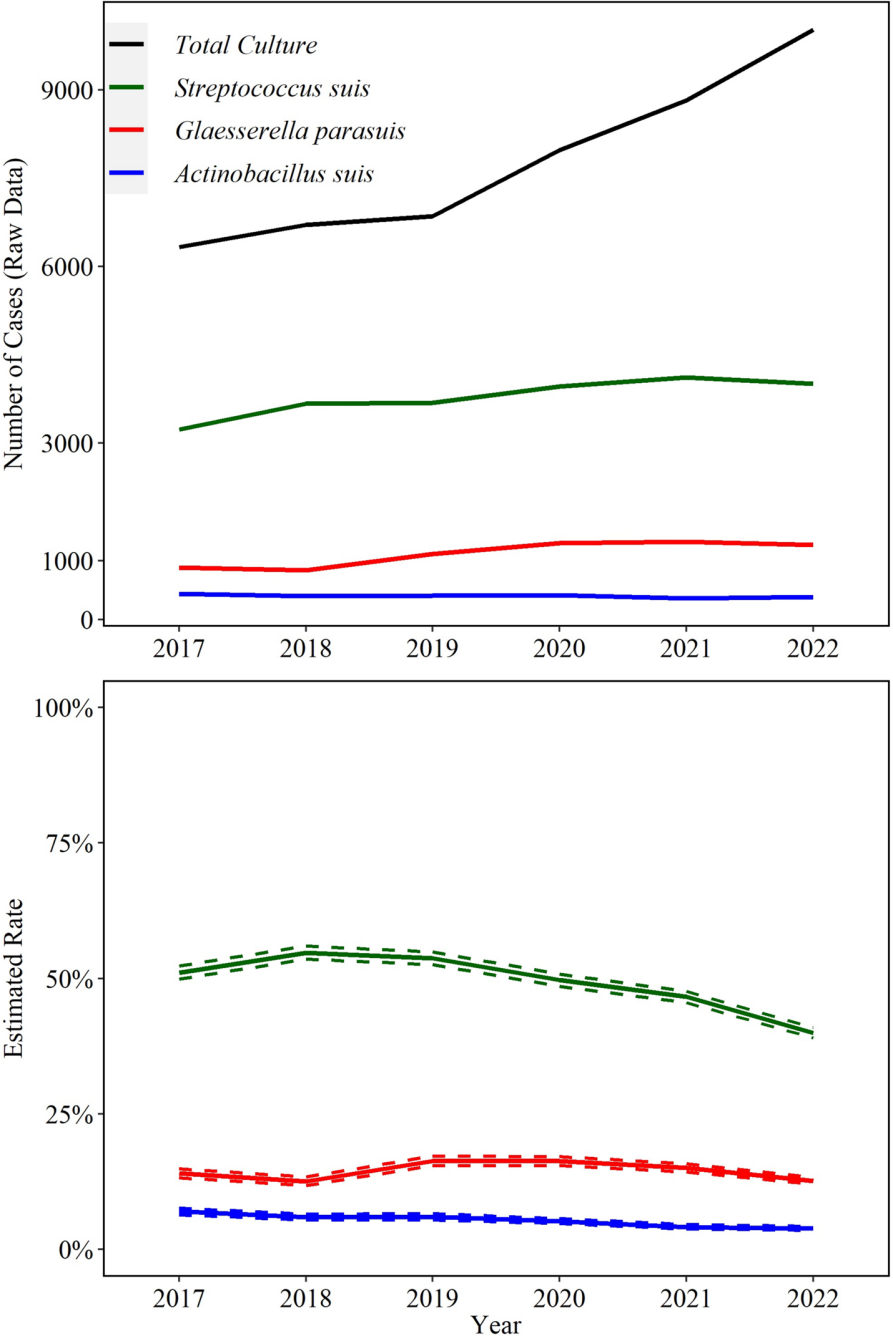
Trendlines (raw data and estimated rate) of overall detection of *Streptococcus suis*, *Glaesserella parasuis*, and *Actinobacillus suis* using bacteriologic culture. The annual estimated rate (solid line) and 95% confidence interval (dashed line) referred to the output of a Binomial regression model which modeled the total number of cases with detection of each agent (colored lines in raw data plot) divided by the total number of bacterial cultures for each year (black line in raw data plot). Black line is based on specimens of interest (see Fig. 2)

A total of 82,563 cases from 2017 to 2022 identified at least one bacterial agent relevant to swine health in any specimen. From those, 26,889 (33%) included isolation of one or more of the three bacterial agents detected by culture included in this study; *S. suis* (22,675; 27%), *G. parasuis* (6753; 8%), and *A. suis* (2429; 3%) were isolated from any specimen. Of these, *S. suis* was isolated from specimens of interest (CNS, heart valve, joint, kidney, liver, lung, serosal fibrin, spleen) in 21,190 (93%) cases, *G. parasuis* was isolated from these specimens in 6612 (98%) cases, and *A. suis* isolation in 2344 (97%) cases Fig 1.

**Fig. 2:**
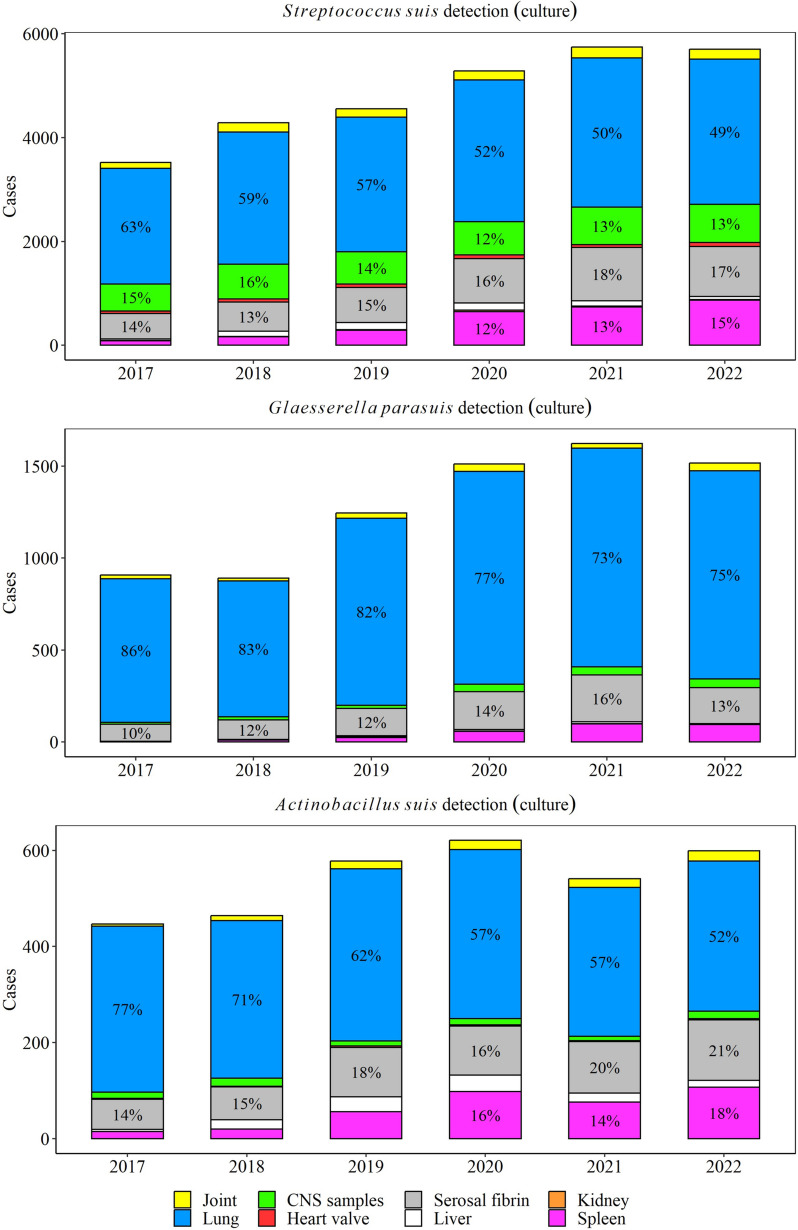
Distribution of specimens with isolation of *Streptococcus suis*, *Glaesserella parasuis*, and *Actinobacillus suis* over a 6-year period. The percent are shown with specimens that were included > 10% of cases

Lungs (50% yearly), serosal fibrin (17% yearly), and CNS (13% yearly) were the most common specimens for *S. suis* isolation, while lungs (75% yearly) and serosal fibrin (13% yearly) were the main specimens for *G. parasuis* isolation. *A. suis* was mostly isolated from lungs (52% yearly), serosal fibrin (20% yearly), and spleen (17% yearly) (Fig. 2).

While the bacterial culture rate of *S. suis* peaked in 2018, this decreased by an average of 9.9% (95% CI 8.9, 11.0%) in the later years (2019–2022, *P* ≤ 0.05). Culture of *A. suis* decreased significantly by an average 12.0% (95% CI 9.7, 14.0%, *P* ≤ 0.05) per year. *G. parasuis* cultures peaked in 2019, but overall the rate did not change in the study period (*P* ≤ 0.05, Fig. 1).

### PCR detection frequencies and trends

**Fig. 3:**
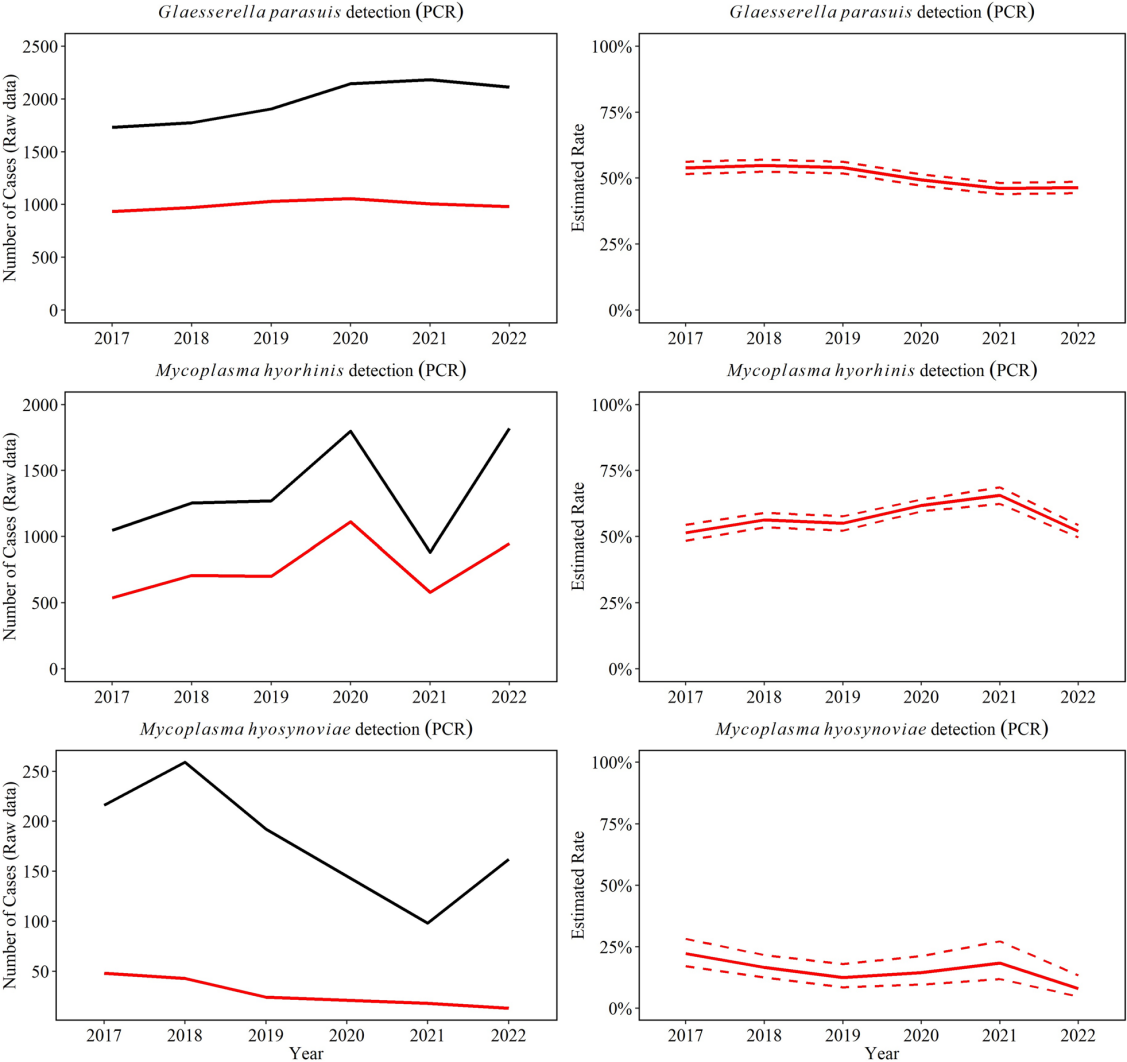
Trendlines (raw data and estimated rate) of overall detection of *Glaesserella parasuis*, *Mycoplasma hyorhinis*, and *Mycoplasma hyosynoviae* using PCR testing in specimens to diagnose disease. The red line represents the positive test results and black line the total testing. The annual estimated rate (solid line) and 95% confidence interval (dashed line) referred to the output of a Binomial regression model which modeled the total number of PCR-positive results from each agent (red line in raw data plot) divided by the total number of PCR tests from each agent for each year (black line in raw data plot)

**Fig. 4:**
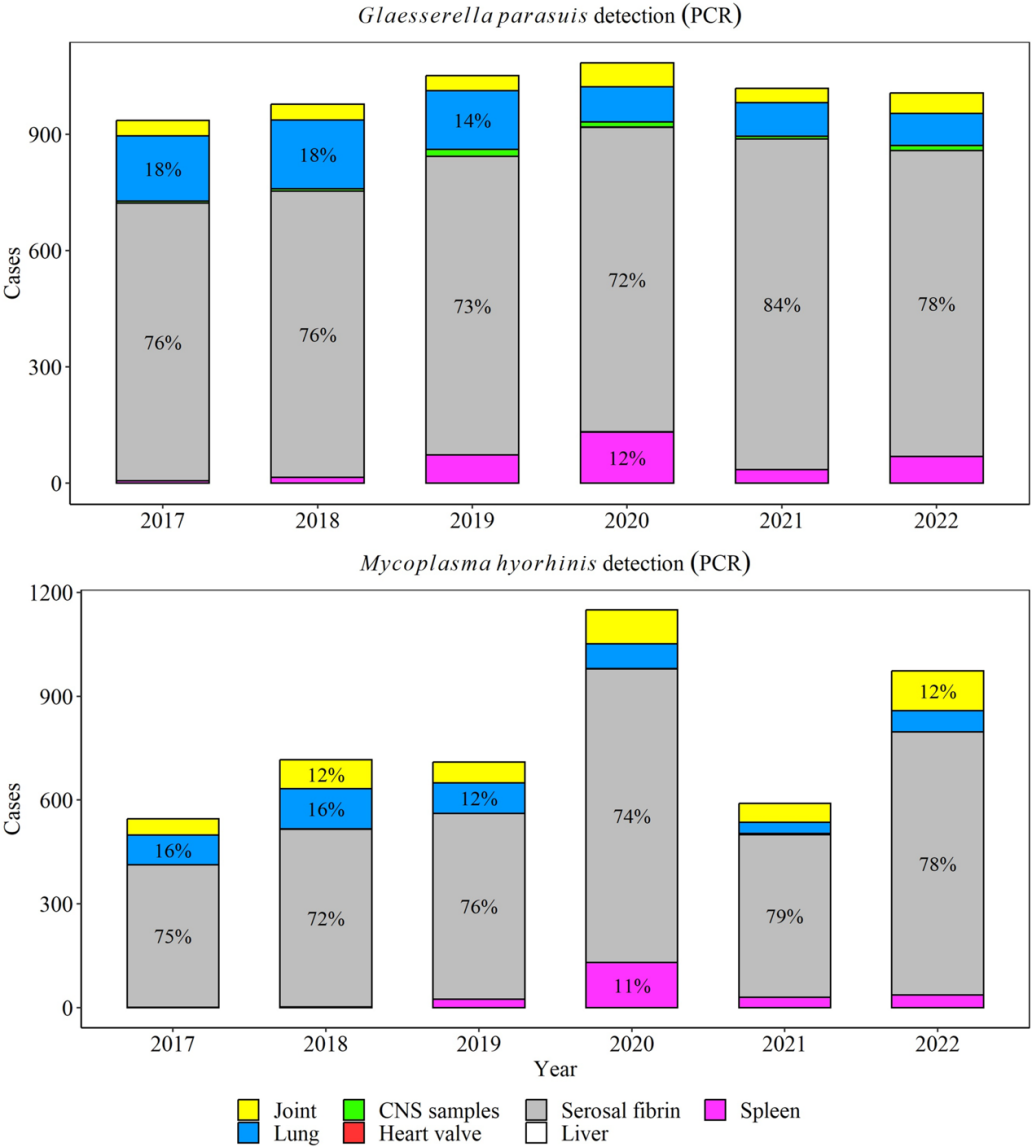
Distribution of specimens with *Glaesserella parasuis* and *Mycoplasma hyorhinis* DNA detection by PCR over a 6-year period. The percent are shown with specimens that were included > 10% of cases

Between 2017 through 2022, a total of 50% (5981/11,849) *G. parasuis* PCR tests were positive in specimens of interest (CNS, heart, serosal fibrin, joint, kidney, liver, lung, and spleen). Overall, there was a significant decrease in the rate of positive PCR results for *G. parasuis* by 0.7% (95% CI 0.5, 0.9%) over the study period (*P* ≤ 0.05, Fig. 3), and the highest detection was observed in 2018. Among *G. parasuis* PCR-positive tests, serosal fibrin (77% yearly) and lungs (12% yearly) were the most common specimens for *G. parasuis* detection (Fig. 4).

*M. hyorhinis* was detected in 57% of all *M. hyorhinis* PCR tests performed (4578 /8069), with serosal fibrin (75% yearly) and joints (10% yearly) being the main specimens tested (Fig. 4). *M. hyorhinis* PCR positivity rate peaked in 2021 (*P* ≤ 0.05, Fig. 3), but overall, it remained steady over the study period (*P* > 0.05).

*M. hyosynoviae* was detected in joints in 22% of all *M. hyosynoviae* PCR tests cases (221/1006) and decreased significantly by an average of 16.0% (95% CI 6.4, 25.0%) per year (*P* ≤ 0.05, Fig. 3).

### Disease diagnosis frequencies

**Fig. 5:**
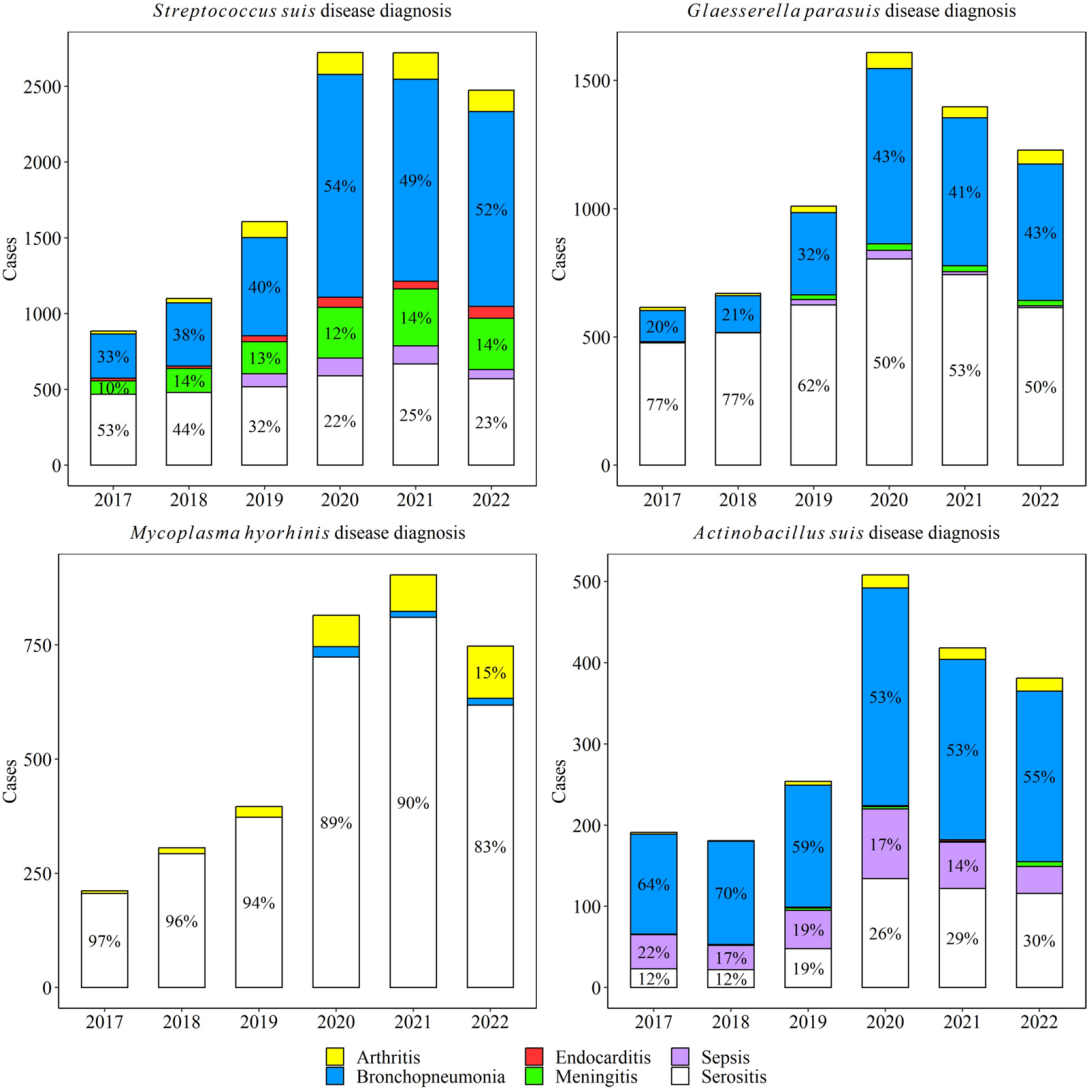
Distribution of lesions observed with *S. suis*, *G. parasuis*, *M. hyorhinis,* and *A. suis* diagnosis over a 6-year period. *M. hyosynoviae* was only observed with arthritis cases. The percent are shown with lesions that were included > 10% of cases

Based on pathologic assessment over 6 years, 44,731 swine cases rendered a disease diagnosis with at least one DxCode with a viral or bacterial infectious etiology. From those, 15,990 (35.7%) cases included disease diagnosis of at least one of the five studied agents. From those cases, 61.7% were *S. suis* (*n* = 9862), 36.0% *G. parasuis* (*n* = 5760), 21.0% *M. hyorhinis* (*n* = 3349), 9.5% *A. suis* (*n* = 1520), and 0.8% *M. hyosynoviae* (*n* = 133). The frequency of these five agents related to the total lesions under disease diagnosis observed in the ISU VDL is shown in Table 1. The annual frequency of lesions associated with systemic infection is shown in Fig. 5. *M. hyosynoviae* was only associated with arthritis cases.

**Table 1:** Frequency of cases with disease diagnosis that included at least one of the five agents by lesion of interest in the whole 6 years of study

Lesion	Total Lesion^1^	*S. suis*		*G. parasuis*		*M. hyorhinis*		*A. suis*		*M. hyosynoviae*	
Arthritis	2069	617	(29.8%)	207	(10.0%)	305	(14.7%)	54	(2.6%)	133	(6.4%)
Bronchopneumonia	16,147	5442	(33.7%)	2379	(14.7%)	51	(0.3%)	1100	(6.8%)	–	–
Endocarditis	509	269	(52.8%)	–	–	–	–	4	(0.8%)	–	–
Meningitis	2136	1508	(70.6%)	92	(4.3%)	–	–	15	(0.7%)	–	–
Sepsis	3396	383	(11.8%)	74	(2.2%)	–	–	295	(8.7%)	–	–
Serositis	17,220	3294	(19.1%)	3779	(21.9%)	3023	(18.8%)	465	(2.7%)	–	–

^1^ Total number of cases that included at least one infectious etiology code (see Supplementary Table 3) with the related lesion

### Disease diagnosis trends

**Fig. 6:**
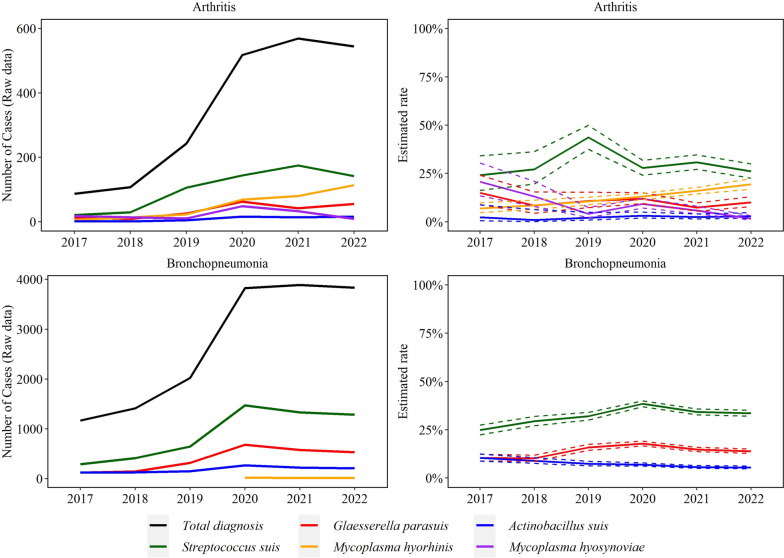
Trendlines (raw data and estimated rate) of arthritis and bronchopneumonia observed with *Streptococcus suis, Glaesserella parasuis*, *Mycoplasma hyorhinis*, *Actinobacillus suis*, and *Mycoplasma hyosynoviae* disease diagnoses. The annual estimated rate (solid line) and 95% confidence interval (dashed line) referred to the output of a binomial regression model which modeled the total number of cases of each agent (colored lines in raw data plot) divided by the total number of cases of the specific lesion for each year (black line in raw data plot)

**Fig. 7:**
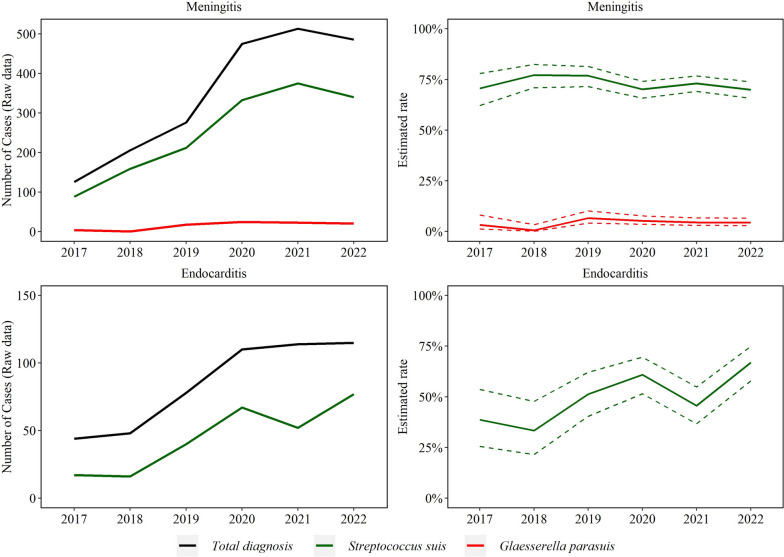
Trendlines (raw data and estimated rate) of endocarditis and meningitis observed with *Streptococcus suis* and *Glaesserella parasuis* disease diagnosis. The annual estimated rate referred to the output of a binomial regression model in which modeled the total number of cases of each agent (colored lines in raw data plot) divided by the total number of cases of the specific lesion for each year (black line in raw data plot)

**Fig. 8:**
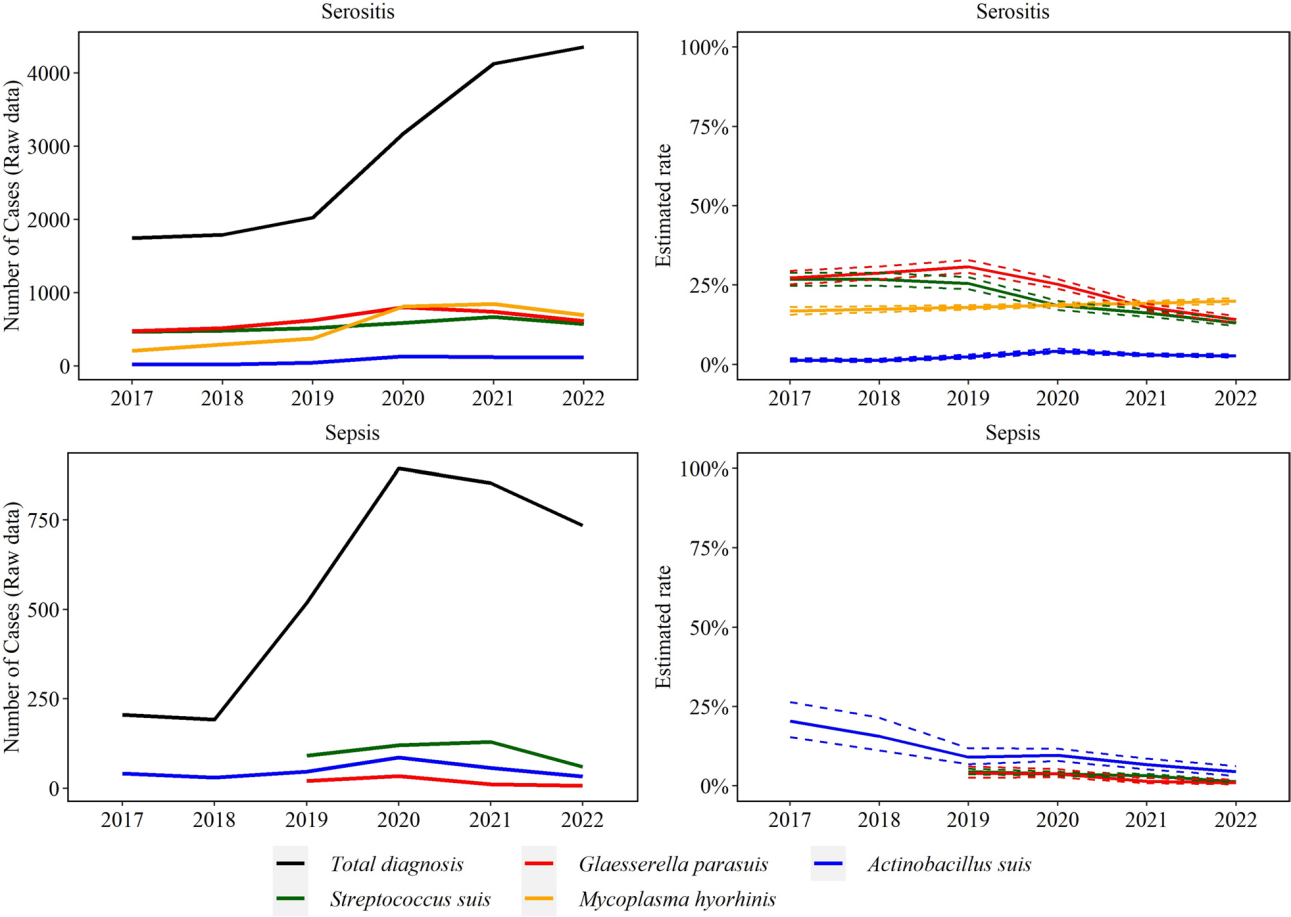
Trendlines (raw data and estimated rate) of sepsis and serositis observed with *Streptococcus suis, Glaesserella parasuis*, *Mycoplasma hyorhinis*, *Actinobacillus suis*, and *Mycoplasma hyosynoviae* disease diagnoses. The annual estimated rate (solid line) and 95% confidence interval (dashed line) referred to the output of a binomial regression model which modeled the total number of cases of each agent (colored lines in raw data plot) divided by the total number of cases of the specific lesion for each year (black line in raw data plot)

There was a significant increase in disease diagnosis for all agents over the study period, except for *M. hyosynoviae,* (Figs. 6-8). Specifically, the rate of *M. hyorhinis* detection in arthritis cases (average 51 cases/year) increased consistently by 26.8% (95% CI 14.9, 40.0%) average yearly for the studied period (*P* ≤ 0.05). In contrast, the rate of *M. hyosynoviae* arthritis (22 cases/year) decreased by 33.0% (95% CI 24.0, 40.0%) on average each year (*P* ≤ 0.05). *S. suis* arthritis peaked in 2019 but overall, no significant trend was detected for *S. suis* (103 cases/year), *G. parasuis* (35 cases/year)*,* or *A. suis* (9 cases/year) in arthritis cases (*P* > 0.05).

The rate of *S. suis* (907 cases/year) and *G. parasuis* (397 cases/year) bronchopneumonia cases increased respectively by 6.0% (95% CI 3.7, 8.3%) and 4.3% (95% CI 0.2, 7.5%) on average each year (*P* ≤ 0.05). In contrast, *A. suis* bronchopneumonia (184 cases/year) decreased by 13.2% on average each year (95% CI 9.8, 16.5%) (*P* ≤ 0.05). A total of 23, 13, and 15 cases of *M. hyorhinis* bronchopneumonia were observed in 2020, 2021, and 2022, respectively.

No significant trend was detected in *S. suis* (252 cases/year) and *G. parasuis* (*n* = 16 cases/year) meningitis (*P* > 0.05). *S. suis* endocarditis (45 cases/year) increased by 23.0% (95% CI 9.8, 38.0%) on average each year (*P* ≤ 0.05, Fig. 7). Trend of *A. suis* endocarditis (4 cases total) and meningitis (15 cases over the study period) were not estimated because of the small sample size.

*S. suis* (101 cases/year)*, G. parasuis* (18 cases/year), and *A. suis* (50 cases/year) sepsis cases decreased over the study period by 30.0% (95% CI 23.0, 36.0%), 41.0% (95% CI 26.0, 54.0%), and 27.0% (95% CI 21.0, 32.0%), respectively (*P* ≤ 0.05).

The rate of *M. hyorhinis* (*n* = 539 cases/year) and *A. suis* (78 cases/year) serositis cases increased by 4.2% (95% CI 1.7, 6.7%) and 12.9% (95% CI 6.4, 19.9%), respectively. *S. suis* (549 cases year) and *G. parasuis* (630 cases/year) cases decreased by an average of 18.0% (95% CI 16.0, 20.0%) and 17.0% (95% CI 16.0, 19.0%) in each year, respectively (*P* ≤ 0.05, Fig. 8).

### Relationship between disease diagnosis and specimens

The denominators for this analysis were cases that included agent detection in specific specimen and histopathology assessment using any specimen. The frequencies of specimens used to assess lesions given that the agent was isolated or detected by PCR are shown in Tables 2 and 3, respectively. For *S. suis*, 72% of isolates from heart valve were given a final diagnosis of *S. suis* endocarditis, 62.1% of isolates from joints were given a final diagnosis of *S. suis* arthritis and 40.3% of isolates from lungs were given a final diagnosis of *S. suis* bronchopneumonia.

**Table 2:** Relationship between disease diagnosis related to a given lesion* and detection by culture (*S. suis, G. parasuis, A. suis*) related to a given specimen by agent. The numerator represents the number of cases with disease diagnosis for the agent associated to a lesion (Example: *S. suis* + Meningitis), and the denominator represent the number of cases in which the agent was detected in a given sample type (Example: *S. suis* + Central Nervous Samples, CNS) but also included a histopathological assessment. These analyses were done for specific lesions and related specimens

** Specimens**	***S*.** ***suis*** **disease diagnosis**					
	**Arthritis**	**Bronchopneumonia**	**Endocarditis**	**Meningitis**	**Sepsis**	**Serositis**
CNS	–	–	–	1443/2994 = 48.1%	–	–
Heart valve	–	–	240/333 = 72.0%	–	–	–
Joint	520/837 = 62.1%	–	–	–	–	–
Kidney	–	–	–	–	12/70 = 17.1%	–
Liver	–	–	–	–	48/443 = 10.8%	–
Lung	–	5199/12,891 = 40.3%	–	–	300/12,891 = 2.3%	–
Serosal fibrin	–	–	–	–	–	2374/4190 = 56.6%
Spleen	–	–	–	–	236/2462 = 9.6%	–
	***G*.** ***parasuis*** **disease diagnosis**					
** Specimens**	**Arthritis**	**Bronchopneumonia**	**Endocarditis**	**Meningitis**	**Sepsis**	**Serositis**
CNS	–	–	–	58/140 = 41.4%	3/140 = 2.1%	62/140 = 44.2%
Joint	98/153 = 64.0%	–	–	–	–	–
Kidney	–	–	–	–	1/7 = 14.3%	3/7 = 42.9%
Liver	–	–	–	–	2/28 = 7.1%	16/28 = 57.1%
Lung	–	2211/5228 = 42.3%	–	–	45/5228 = 0.8%	1441/5228 = 27.6%
Serosal fibrin	–	–	–	–	20/936 = 2.1%	818/936 = 87.4%
Spleen	–	–	–	–	23/266 = 8.7%	187/266 = 70.3%
	***A*.** ***suis*** **disease diagnosis**					
** Specimens**	**Arthritis**	**Bronchopneumonia**	**Endocarditis**	**Meningitis**	**Sepsis**	**Serositis**
CNS	–	–	–	14/54 = 25.9%	6/54 = 11.1%	5/54 = 9.2%
Heart valve	–	–	4/12 = 33.3%	–	–	–
Joint	52/67 = 77.6%	–	–	–	–	–
Kidney	–	–	–	–	9/16 = 56.2%	–
Liver	–	–	–	–	57/113 = 50.4%	31/113 = 27.4%
Lung	–	1006/1651 = 60.9%	–	–	265/1651 = 16.0%	353/1651 = 21.8%
Serosal fibrin	–	–	–	–	69/526 = 13.2%	290/526 = 55.1%
Spleen	–	–	–	–	141/344 = 40.9%	126/344 = 36.7%

*The number of cases with lesions (numerator) differs from Table 1 because not all cases reported a specimen or possibly a testing (culture or PCR) in the bacteriology section or molecular section

**Table 3:** Relationship between disease diagnosis related to a given lesion* and detection by PCR (*G. parasuis*, *M. hyorhinis*, *M. hyosynoviae*) related to a given specimen by agents. The numerator represents the number of cases with disease diagnosis for the agent associated to a lesion (Example: *M. hyorhinis* + Serositis), and the denominator represent the number of cases in which the agent was detected in a given sample type (Example: *M. hyorhinis* + Positive+Serosal fibrin, SF) and histopathology was performed. These analyses were done for specific lesions and related specimens

**Specimens**	***G*.** ***parasuis*** **disease diagnosis**				
	**Arthritis**	**Bronchopneumonia**	**Meningitis**	**Sepsis**	**Serositis**
CNS	–	–	20/47 = 42.5%	–	8/47 = 17.0%
Joint	124/230 = 53.9%	–	–	–	–
Liver	–	–	–	1/1 = 100%	–
Lung	–	105/464 = 22.7%	–	2/464 = 0.4%	53/464 = 11.4%
Serosal fibrin	–	–	–	27/4338 = 0.4%	3145/4338 = 72.5%
Spleen	–	–	–	22/282 = 7.8%	100/282 = 35.4%
	***M*.** ***hyorhinis*** **disease diagnosis**				
**Specimens**	**Arthritis**	**Bronchopneumonia**	**Meningitis**	**Sepsis**	**Serositis**
CNS	–	–	–	–	–
Joint	226/392 = 57.7%	–	–	–	–
Lung	–	36/293 = 12.3%	–	–	–
Serosal fibrin	–	–	–	–	2471/3368 = 73.4%
Spleen	–	–	–	–	–
	***M*.** ***hyosynoviae*** **disease diagnosis**				
**Specimens**	**Arthritis**	**Bronchopneumonia**	**Meningitis**	**Sepsis**	**Serositis**
Joint	133/160 = 83.1%	–	–	–	–

*The number of cases with lesions (numerator) differs from Table 1 because not all cases reported a specimen or possibly a testing (culture or PCR) in the bacteriology section or molecular section

For *G. parasuis,* 87.4% of isolates and 72.5% of PCR detections from serosal fibrin (such as fibrin, liver surface, lung surface, spleen surface, pleura, heart, etc.) were associated with a final diagnosis of *G. parasuis* serositis, while 42.3% of *G. parasuis* isolates and 22.7% of PCR detections from the lung were associated with bronchopneumonia. Approximately 41% of *G. parasuis* isolates from CNS specimens were given a final diagnosis of *G. parasuis* meningitis. Similarly, 42.5% of *G. parasuis* PCR-positive CNS specimens were given a final diagnosis of *G. parasuis* meningitis.

For *A. suis*, 77.6 and 60.9% of isolates recovered from joints and lungs were given a final diagnosis of *A. suis* arthritis and bronchopneumonia, respectively. Like *G. parasuis,* 73.4% of *M. hyorhinis* PCR-positive serosal fibrin tests were given a final diagnosis of *M. hyorhinis* serositis. Finally, 83.1% of *M. hyosynoviae* PCR-positive joint samples were diagnosed as *M. hyosynoviae* arthritis.

### Association between pig production phase and specific agent-associated lesions

**Fig. 9:**
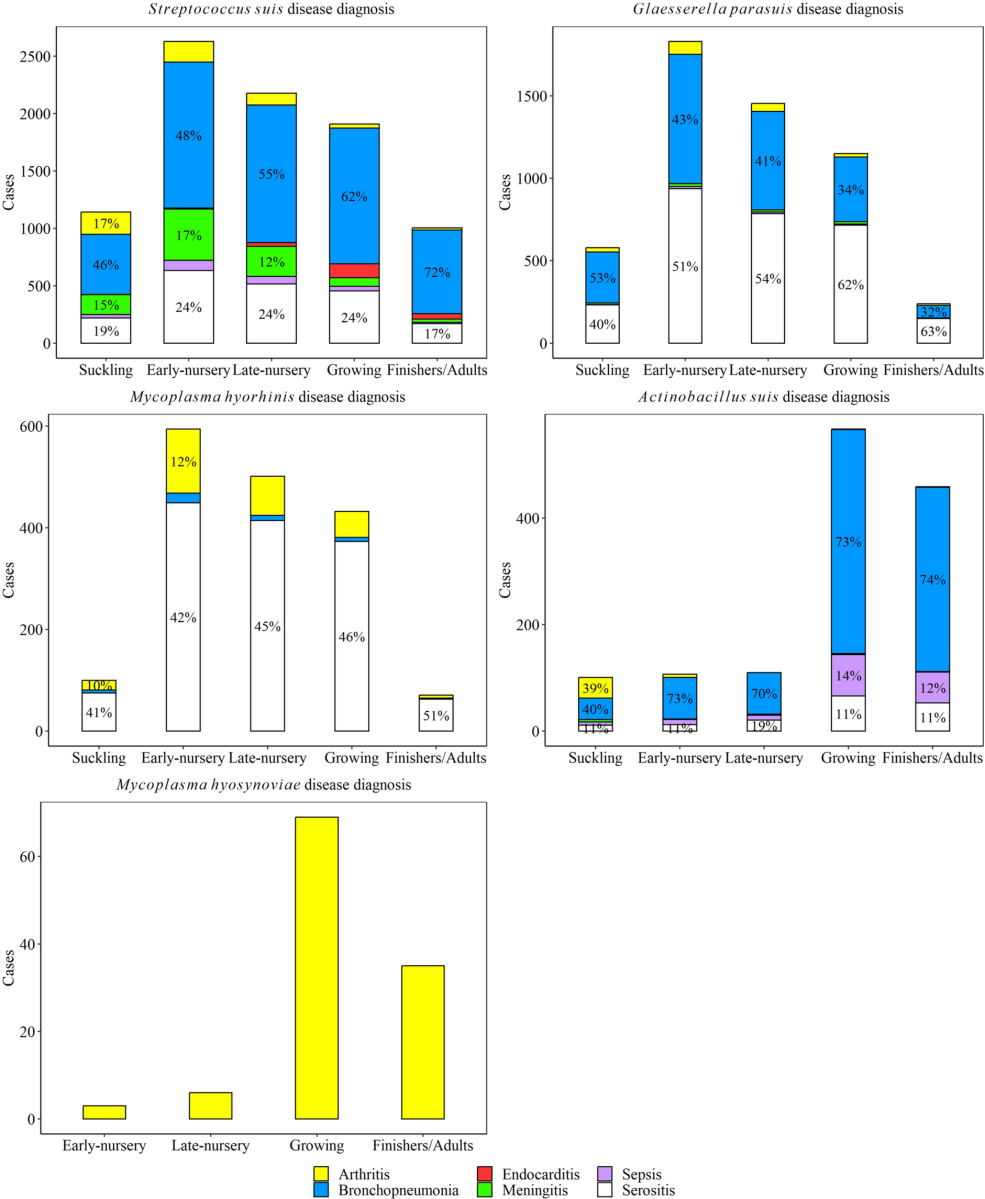
Distribution of lesions observed with *Streptococcus suis, Glaesserella parasuis, Mycoplasma hyorhinis*, *Actinobacillus suis*, and *Mycoplasma hyosynoviae* diseases by age of pigs. Suckling piglets (0 < x ≤ 3-week-old); early-nursery (3 < x ≤ 6-week-old); late-nursery (6 < x ≤ 10-week-old); growing (10 < x ≤ 16-week-old); and finishers and adults (16-week-old < x). The percent are shown with pig production phases that were included > 10% of cases

The distribution of pig production phase by lesion for all five agents is shown in Fig. 9. *S. suis* arthritis cases were higher in suckling and early-nursery pigs, while *S. suis* bronchopneumonia disease was higher in early-, late-nursery, and growing pigs (*P* ≤ 0.05). *S. suis* endocarditis was highest in the growing phase, while *S. suis* meningitis was highest in early-nursery compared to all other phases (*P* ≤ 0.05). Sepsis and serositis cases due to *S. suis* were statistically higher in early-nursery, late-nursery, and growing pigs than in the suckling and finishing pigs (*P* ≤ 0.05).

*G. parasuis* arthritis and bronchopneumonia cases were statistically higher in early- and late-nursery compared to other phases (*P* ≤ 0.05). *G. parasuis* meningitis was higher in suckling, early-, and late-nursery pigs than in growing and finishing pigs (*P* ≤ 0.05). No statistical difference was observed in *G. parasuis* sepsis cases among pig production phases (*P* > 0.05). *G. parasuis* serositis cases were higher in early-nursery, compared to late-nursery and growing phases (*P* ≤ 0.05).

*M. hyorhinis* arthritis and serositis were higher in early-, late-nursery, and growing phases (*P* ≤ 0.05). *A. suis* arthritis cases were statistically higher in suckling pigs, while bronchopneumonia, sepsis, and serositis were all statistically higher in growing and finishing pigs compared to other phases (*P* ≤ 0.05). *M. hyosynoviae* arthritis cases were higher in finishing and adult pigs (*P* ≤ 0.05).

### Polymicrobial diagnosis

As shown in Table 4, disease diagnoses of all five agents were most frequently observed with other infectious etiologies. The most common viral agent co-detected with disease caused by *S. suis, G. parasuis, A. suis* and *M. hyorhinis* was PRRSV, followed by IAV. When combined with other infectious etiologies, *S. suis, G. parasuis, M. hyorhinis,* and *A. suis* disease diagnoses were substantially higher compared to diagnosis on their own. *M. hyosynoviae* cases were more frequently diagnosed without any other etiology.

**Table 4:** Number of disease diagnosis cases that included at least one of the five bacterial agents (and the most common polymicrobial interactions within a case) by body system in the last 6 years

Infectious etiology(ies)	Total	%^1^	Annual Average	Body system^2^
***S. suis cases***	**9868**	**100%**		
*S. suis* (only)	2511	25%	419	Mult Sys (55%), Res (22%), Ner (14%), Mus (5%), Card (4%)
*S. suis* + any other infectious etiology	7357	75%	–	Mult Sys (72%), Res (23%)
*S. suis* + PRRSV	962	10%	160	Mult Sys (79%), Res (21%)
*S. suis* + *G. parasuis*	458	5%	77	Mult Sys (76%), Res (24%)
*S. suis* + IAV	374	4%	63	Res (64%), Mult Sys (36%)
*S. suis* + *G. parasuis* + PRRSV	326	3%	39	Mult Sys (87%), Res (13%)
*S. suis* + *multocida* + PRRSV	313	3%	31	Mult Sys (73%), Res (27%)
*S. suis* + *multocida*	228	2%	38	Res (75%), Mult Sys (25%)
*S. suis* + IAV + PRRSV	175	2%	29	Mult Sys (73%), Res (27%)
*S. suis* + *G. parasuis* + *M. hyorhinis* + PPRSV	167	2%	28	Mult Sys (98%), Res (2%)
*S. suis* + *G. parasuis* + IAV	137	1%	23	Res (52%), Mult Sys (48%)
*S. suis* + *M. hyorhinis* + PPRSV	137	1%	23	Mult Sys (99%), Res (1%)
***G. parasuis*** **cases**	**5760**	**100%**		
*G. parasuis* (only)	941	16%	157	Mult Sys (63%), Res (30%), Card (3%), Mus (2%), Ner (1%)
*G. parasuis* + any other infectious etiology	4819	84%	–	Mult Sys (79%), Res (21%)
*G. parasuis* + PRRSV	531	9%	89	Mult Sys (84%), Res (16%)
*G. parasuis* + IAV	299	5%	50	Res (49%), Mult Sys (51%)
*G. parasuis* + *M. hyorhinis* + PRRSV	183	3%	31	Mult Sys (98%), Res (2%)
*G. parasuis* + *M. hyorhinis*	169	3%	29	Mult Sys (90%), Res (5%), Mus (5%)
*G. parasuis* + IAV + PRRSV	133	2%	23	Mult Sys (76%), Res (24%)
***M. hyorhinis*** **cases**	**3292**	**100%**		
*M. hyorhinis* (only)	281	9%	55	Mult Sys (85%), Mus (8%), Card (4%), Res (2%)
*M. hyorhinis* + any other infectious etiology	3011	91%	6	Mult Sys (87%), Res (13%)
*M. hyorhinis* + PRRSV	345	10%	60	Mult Sys (96%), Res (4%)
***A. suis*** **cases**	**1831**	**100%**		
*A. suis* (only)	682	37%	114	Res (52%), Mult Sys (41%), Mus (4%), Card (2%)
*A. suis* + any other infectious etiology	1149	63%	–	Mult Sys (70%), Res (29%)
*A. suis* + PRRSV	121	7%	20	Mult Sys (62%), Res (38%)
***M. hyosynoviae cases***	**133**	**100%**		
*M. hyosynoviae* (only)	95	71%	7	Mus (100%)
*M. hyosynoviae* + any other infectious etiology	38	29%	2	Mus (98%), Mult Sys (2%)
*M. hyosynoviae +* PRRSV	6	5%	2	Mult Sys (100%)
*M. hyosynoviae + S. suis*	4	3%	1	Mus (75%), Mult Sys (25%)

^1^Percentages are based on the total number of cases of the five bacterial agents of interest

^2^Card Cardiovascular-Blood-Endocrine-Immune, Mus Musculoskeletal, Mult Sys Multisystemic, Ner Nervous, Res Respiratory

## Discussion

The impact of *S. suis, G. parasuis, M. hyorhinis, A. suis, and M. hyosynoviae* is widespread throughout the swine production chain, resulting in detrimental effects on pig health and welfare, decreased productivity, and increased production costs. Disease control measures currently involve enhancing immunity through gilt acclimation or vaccination, mitigating known management and environmental risk factors, and implementing judicious antimicrobial use. However, due to the global decline in prophylactic and metaphylactic use of antimicrobials in the swine industry, production systems must refine prevention strategies to reduce their dependence on antimicrobials. Therefore, it becomes crucial to accurately and promptly diagnose these complex endemic agents to gain valuable insights into disease occurrence and presentation. To date, temporal detection and disease diagnosis data have been scarcely described in the literature for these five endemic bacteria. Trevisan et al. [26] described the value of robust and standardized datasets, containing diagnostic data stored at U.S. VDLs, and how they can be applied to understand PRRSV occurrence at the regional level. Thus, this study aimed to determine temporal diagnostic trends of five endemic bacterial agents from field cases using 6-years of data stored at the ISU VDL.

Establishing the disease occurrence of these endemic agents at the herd level can be problematic because of their commensal ecology, concurrent infections, and broad spectrum of clinical signs and lesions [3, 19]. Their commensal status in the respiratory tract precludes using antemortem specimens (e.g., nasal swab, oral fluid, tracheal wash, bronchiolar lavage fluid) to solely determine causation. Furthermore, detecting these agents in upper respiratory tract samples (e.g., tonsil, nasal or tracheal samples) does not necessarily imply disease. These sample types are more commonly employed in agent surveillance and disease monitoring for viral and some bacterial agents [27]. Thus, disease diagnosis often requires isolation or detection in specific tissues with compatible lesions. This study used field cases containing a predefined set of specimens where these agents are more likely to be related to the clinical outcome.

There was an overall numerical and sometimes statistical increase in the detection and disease diagnosis rates for all agents in the last 6 years, except for *M. hyosynoviae*. The reason for this increase is likely multifactorial. From a data analysis perspective, the findings may reflect the recent optimization in data management in the ISU VDL. For example, ISU VDL improved the standards for DxCode in 2019 with changes in the DxCode format, setting a new standard and clarification of diagnostic coding among the diagnosticians [20, 25]. This change could have a role in the increase in the number of some disease diagnoses, such as bronchopneumonia, from 2019 to 2022 observed in this study. From an industry perspective, the growth of the swine industry observed between 2015 and 2019 (550,000 more sows that were younger and less-immune were added to the U.S. breeding herd inventory) [28], and/or industry-wide management changes (e.g., reduction in antimicrobial use) [29, 30] could have contributed to the noted increase in the sample submission and disease diagnosis. From a surveillance, disease management, and diagnostic perspective, the increase could also be due to amplified awareness of the impact of these agents and the advancements in diagnostic techniques, e.g., novel PCRs and whole genome sequencing implemented in diagnostic laboratories [7, 31]. The increased number of cases including a specific sample type (e.g., spleens) for agent detection might be due to the collection of that sample type for African Swine Fever and Classical Swine Fever surveillance purposes [32], thus resulting in the increased detection and disease diagnosis of these bacterial agents in recent years.

The increase could also reflect a growing interest by the swine industry in more in-depth testing, such as serotyping, whole genome sequencing, antimicrobial susceptibility testing, and the need for bacterial isolates for autogenous vaccine production. Finally, fluctuations in agent detection trends might also be due to changes in the prevalence of the agent or the dissemination of more pathogenic variants. *S. suis* sequence type 1 strains, a genotype considered of high pathogenicity and previously thought to be mainly found in European and Asian countries, has been more often detected in U.S. swine herds [33]. Similarly, serotype 7 *G. parasuis* strains, previously considered non-pathogenic, have recently been described as the most frequently detected genotype associated with disease [34, 35]. Outbreaks of viral diseases, commonly recognized as catalysts for endemic bacterial diseases, exemplified by the PRRSV-2 variant within lineage 1C diseases, could have also contributed to the observed increase [36]. Still, the precise causes of the observed increases or decreases need further elucidation.

Among the five studied agents, *S. suis* was the most frequently detected and diagnosed agent. In fact, 22% of all cases with infectious etiology (bacteria or viruses) included a *S. suis* disease diagnosis, and 27% of all bacteriology cases included *S. suis* isolation. Furthermore, although a trend was not detected, it was the predominant pathogen in neurological cases submitted to the ISU VDL (70.6% of all cases). Within nervous cases, *S. suis* disease was more frequently diagnosed without other infectious etiologies. *S. suis* diagnosis was regularly diagnosed in suckling and nursery phases of pig production, regardless of the lesion, as reported previously [37]. Results of this study also show that nearly half of the cases with a *S. suis* diagnosis occurred in pigs in the suckling, growing or finisher/adult stages, highlighting the impact of this agent across all pig production phases. The number of cases of *S. suis* meningitis disease was higher in suckling and early-nursery (up to 6-week-old) than late-nursery pigs, correlating with previous reports [38, 39]. This study also highlighted the significant upward trend in *S. suis* endocarditis in growing and finisher pig cases. Taken together, these findings highlight the significant role *S. suis* plays in meningitis, arthritis and serositis in younger pigs as a primary pathogen, and endocarditis in older pigs.

These data also showed constant upward trend in *S. suis* bronchopneumonia cases in the last 6 years; however, within bronchopneumonia cases, *S. suis* was co-detected with other agents, such as *G. parasuis*, *M. hyorhinis*, IAV, and PRRSV. Therefore, the significant increase of *S. suis* bronchopneumonia might also by explained by the increase of primary viruses (IAV and PRRSV). These observations align with previous reports on the role of *S. suis* as a secondary pathogen in respiratory disease cases [10]. This study demonstrated that *S. suis* played a role within multi systemic cases along with other infectious pathogens, e.g., *S. suis* disease cases were often found as unique diagnosed etiology and interacting with primary viruses and other bacteria.

*G. parasuis* was the second most frequently detected and diagnosed agent and one of the most diagnosed bacterial agents in serositis cases (21.9% of all cases) over the study period. It is also diagnosed in 10% of all infectious arthritis cases. Trends analyses revealed that *G. parasuis* serositis increased from 2017 until 2020, followed by a downward trend between 2020 and 2022. Similarly, to *S. suis, G. parasuis* bronchopneumonia increased significantly in the last 6 years, which could reflect the simultaneous increasing diagnosis of other etiologies, such as PRRSV. Co-diagnosis of *G. parasuis* with other infectious etiologies, such as PRRSV, IAV, and *M. hyorhinis,* was more frequent in respiratory and systemic cases (Table 4). Finally, *G. parasuis* disease was diagnosed mostly in the nursery phase of production, which has been previously reported [11, 40]. Thus, given the frequency of *G. parasuis* in nursery polymicrobial systemic disease cases, it implies that *G. parasuis* control likely hinges on the management of other bacterial and viral co-infections.

Trends analyses showed increased *M. hyorhinis* detection, mainly from 2018 through 2020, and a significant increase in *M. hyorhinis* serositis and arthritis cases over the study period. Specifically, 19% of all serositis and 14% of all arthritis cases were given a *M. hyorhinis* diagnosis (Table 1). Interestingly, Table 4 shows that *M. hyorhinis* disease was more commonly co-diagnosed with *S. suis, G. parasuis,* and PRRSV as previously reported [41–43]. In fact, only 8.5% of cases with an *M. hyorhinis* diagnosis, included only this agent. In the cases of arthritis, *M. hyorhinis* was more commonly diagnosed on its own (Table 4). While no significant trend was observed, there was a numerical increase in *M. hyorhinis* bronchopneumonia in the last 3 years. In fact, in situ hybridization with RNAscope® has recently been used to identify *M. hyorhinis* within lesions that were commonly associated with *M. hyopneumoniae* [44]. Still, the role of *M. hyorhinis* in this lesion is poorly understood [43]. *M. hyorhinis* disease was mostly diagnosed in nursery and growing pigs highlighting this agent’s the relevance in post-weaning systemic disease [45].

Disease associated with *A. suis* is mainly observed in cases of septicemia in suckling or recently weaned pigs, or in grow-finish pigs experiencing bronchopneumonia and serositis [6]. The similarities in clinical presentation with *A. pleuropneumoniae* disease complicates accurate *A. suis* diagnosis [46, 47]. In this study, *A. suis* detection and disease diagnosis were less frequent compared to *S. suis, G. parasuis* and *M. hyorhinis.* However, there was a significant increase in cases of *A. suis* serositis over the study period*,* aligning with recent reports [48]. Furthermore, *A. suis* was diagnosed in 8.7% of all sepsis cases, mainly in growing and finishing pigs. In contrast, a decreasing trend of *A. suis* bronchopneumonia and arthritis was observed which might indicate suboptimal surveillance efforts for this pathogen or simply reflect its opportunistic nature. Additionally, cases identifying *A. suis* arthritis were mainly seen in suckling piglets 2021 (*n* = 13), 2020 (*n* = 12 cases), and 2019 (*n* = 2) (Fig. 9), and not in later stages of production, suggesting that *A. suis* is an agent to consider in pre-weaning polyarthritis. *A. suis* disease diagnosis including bronchopneumonia and systemic infections were statistically associated with later phases of production in this study, correlating with previous knowledge [48]. Finally, *A. suis* disease as the sole etiology represented 37% of all cases with an *A. suis* diagnosis, suggesting a primary role in swine disease.

Regardless of the overall increase in arthritis cases observed during the study period (Fig. 6), *M. hyosynoviae* arthritis diagnosis significantly decreased over time. Notably, the number of cases with PCR testing for *M. hyosynoviae* also decreased (Fig. 4). *M. hyosynoviae* infection was found more often in finisher and adult pigs, as reported previously [18, 49, 50]. While data from this study show that *M. hyosynoviae* diagnosis decreased over the study period, reports from the field have shown that infectious lameness is a common occurrence in grow-finish herds and may play a role in sow mortality [51, 52]. The reason for the decline in diagnosis of *M. hyosynoviae* arthritis might be due to a decline in the submission of arthritis cases in adult pigs, since *M. hyosynoviae* impacts later stages of production and can be controlled via the use of antimicrobials, or it could reflect the complexity of diagnosing this agent under field settings [53]. Though there are many non-infectious and infectious causes of lameness, future epidemiology studies that include data from multiple laboratories are needed to understand the frequency of *M. hyosynoviae* lameness in the field.

Overall, tissues and specimens used to detect and diagnose disease of each of the five agents were submitted in accordance with their previously described pathology [19, 54, 55]. *S. suis, G. parasuis,* and *A. suis* were mainly detected through bacteriological culture using lung samples. However, from 2020 other systemic tissues were more frequently used for detection, such as CNS samples (i.e., brain, brain swab, cerebrospinal fluid), serosal fibrin, and spleen. Given that the lung may harbor a wide range of commensal bacteria [54, 56], it is critical that the diagnosis of disease is based not solely on detection data but also in association with the pathological changes. Thus, this study provided insights into which specimens were most used for disease diagnosis based on the histopathology evaluation with agent detection data. For instance, 69.6% (15,786/22,675) of all *S. suis* isolates originated from lungs. However, 40.3% of those lung isolates were used to diagnose *S. suis* bronchopneumonia cases. Only 4% of all *S. suis* isolates originated from joints (1025/22,675), but from those, 62.1% were given *S. suis* arthritis disease diagnosis. Similarly in neurological cases, 17.2% (3899/22,675) of *S. suis* isolates originated from CNS samples, and 48.1% of those were used to diagnose meningoencephalitis. These data imply that a significant proportion of isolates (52%) obtained from CNS samples originate from cases that, either do not have enough evidence for diagnosis (i.e., tissues were not submitted, only meningeal swabs or cerebrospinal fluid), or histopathological lesions were not observed, indicating potential cross-contamination of the sample. These data highlight the importance of proper animal selection, sample collection and handling for disease diagnosis [19]. Overall, isolation from lungs was less predictive of *S. suis* disease diagnosis compared to joints, serosal fibrin, and heart valves. Similar findings were observed with G. parasuis. While 89% (6015/6753) of all *G. parasuis* isolates were obtained from the lung, 42.3% were used to diagnose *G. parasuis* bronchopneumonia. Around 15% of all *G. parasuis* isolates were obtained from serosal fibrin samples, but from those isolations, 87.4% were used to give a *G. parasuis* serositis diagnosis, highlighting the diagnostic value of serosal fibrin for *G. parasuis* diagnosis. Assessment of clinical history, gross and microscopic changes, and isolation or detection by PCR in specific sites (CNS samples, joints, serosal fibrin, and spleen), coupled with additional diagnostic tests (serotyping and sequencing), are critical for accurate assessment of *S. suis-* and *G. parasuis-*associated diseases. Great care should be taken when collecting tissues for diagnostic submission, as *S. suis* or any other of the four bacterial agents are commensals that can easily confound culture or PCR results by contaminating lesioned tissues [57].

Like other studies based on diagnostic data [25, 27], caution is needed when interpreting the findings from this study, which used three large datasets created based on specific information from diagnostic cases stored in a data warehouse at a single diagnostic laboratory. Therefore, results from either detection and disease diagnosis depended on what was submitted (case and selection bias) and what was targeted by the submitter (cognitive biases) but also what information was defined as relevant by the group of diagnosticians from the ISU VDL. For instance, while these five agents can be considered secondary pathogens in the infectious process, they can also contribute to some of the observed lesions with or without other risk factors (infections/noninfectious). Thus, the DxCodes used in this study suggested what the cause of the lesion is but may not necessarily reflect main problem in the clinical context.

Additionally, true prevalence and incidence of the five agents cannot be assessed given that there is no randomness in sample collection or presence of true negative cases. Yet, this study data suggested a consistent increase in efforts to detect and diagnose diseases associated with the five endemic bacterial agents investigated and improved disease diagnostic codes. Furthermore, implementation of veterinary diagnostic codes for disease diagnosis monitoring is a recent achievement at the ISU VDL [20].

## Conclusions

Understanding diagnostic trends for the primary drivers of antimicrobial use in swine farms is crucial, considering the swine industry’s emphasis on antimicrobial stewardship. This retrospective study utilized a comprehensive dataset (i.e., ~ 27 k bacteriological cases and ~ 16 k histopathological reports) collected over a 6-year period. Results from this study demonstrated significant increases in disease diagnosis for *S. suis*, *G. parasuis*, *M. hyorhinis,* and *A. suis*, and a significant decrease in detection and disease diagnosis of *M. hyosynoviae*. Investigation into the diagnostic value of particular tissues showed that lungs were frequently included for detection and disease diagnoses. However, the use of lung for systemic disease diagnosis requires caution due to the commensal nature of these agents in the respiratory system, compared to systemic sites that diagnosticians typically target. Bacterial isolation and DNA in systemic sites did not necessarily indicate the presence of disease caused by these five agents. For instance, half of *S. suis* isolation in CNS samples was used in meningitis diagnoses associated with *S. suis* infections. Thus, the anatomic location selected for sampling from acutely affected, nonmedicated animals, based on a case definition and proper diagnostic testing, gross lesions, and verified compatible histopathologic lesions, offers a comprehensive assessment for the contribution of these bacteria to systemic disease. Given that commercial vaccines are not available for the majority of these pathogens, it is essential to conduct a thorough diagnostic process to improve the likelihood of obtaining disease-associate strain candidates for the development of autogenous vaccines.

This study also explored associations between phase of production and specific diseases caused by each agent, showcasing the role of *S. suis* arthritis in suckling pigs, meningitis in early nursery and endocarditis in growing pigs, and the role of *G. parasuis, A. suis, M. hyorhinis* and *M. hyosynoviae* disease mainly in post-weaning phases. Hence, it is necessary to take into consideration the onset of disease throughout the various phases of pig production when formulating control strategies for these four agents. Finally, this study highlighted the high frequency of co-detection with other infectious etiologies, such as PRRSV and IAV, demonstrating that to minimize the health impact of these endemic bacterial agents it is imperative to establish effective viral control programs.

### Supplementary Information

**Additional file 1.**

## Data Availability

The datasets presented in this article are not readily available because Client confidentiality is strictly maintained, but the data can be searched and recovered for analysis by authorized personnel only. Requests to access the datasets should be directed to Maria Jose Clavijo, mclavijo@iastate.edu.

## Abbreviations

ISU VDL: Iowa State University Veterinary Diagnostic Laboratory
LIMS: Laboratory Information Management System
PCR: Polymerase chain reaction
DxCodes: Disease diagnostic codes
CNS: Central nervous samples
PRRSV: Porcine reproductive and respiratory syndrome virus
IAV: Influenza a vírus

## References

[1] Swine health information center. Swine bacterial disease matrix [internet]. 2021 [cited 2023 Jan 9]. Available from: https://www.swinehealth.org/swine-bacterial-disease-matrix/

[2] Hayer SS, Rovira A, Olsen K, Johnson TJ, Vannucci F, Rendahl A (2020). Prevalence and time trend analysis of antimicrobial resistance in respiratory bacterial pathogens collected from diseased pigs in USA between 2006–2016. Res Vet Sci..

[3] Burrough ER, Baum DH, Schwartz KJ (2019). Collecting evidence and establishing causality. Diseases of swine.

[4] Aragon V, Segalés J, Tucker AW (2019). Glässer’s Disease. Diseases of Swine.

[5] Gottschalk M, Segura M (2019). Streptococcosis. Diseases of swine.

[6] Gottschalk M, Broes A (2019). Actinobacillosis. Diseases of swine.

[7] Clavijo M, Mugabi R, Ganwu L. Friend or foe: what next generation sequencing can tell you about the endemic agents in your herd. In: Proceedings of the 52nd Annual Meeting of the American Association of Swine Veterinarians. San Francisco; 2021. p. 376–7.

[8] Dial G, Rademacher C, Wiseman B, Roker J, Freking B. Costs, consequences and control of endemic diseases. In: 2nd London swine conference. London and Ontario; 2002.

[9] Wei YW, Zhu HZ, Huang LP, Xia DL, Wu HL, Bian HQ (2020). Efficacy in pigs of a new inactivated vaccine combining porcine circovirus type 2 and *mycoplasma hyorhinis*. Vet Microbiol..

[10] Obradovic MR, Segura M, Segalés J, Gottschalk M (2021). Review of the speculative role of co-infections in *Streptococcus suis*-associated diseases in pigs. Vet Res..

[11] Oliveira S, Pijoan C, Morrison R (2004). Evaluation of *Haemophilus parasuis* control in the nursery using vaccination and controlled exposure. J Swine Health Prod..

[12] Giménez-Lirola LG, Meiroz-De-Souza-Almeida H, Magtoto RL, McDaniel AJ, Merodio MM, Matias Ferreyra FS (2019). Early detection and differential serodiagnosis of *mycoplasma hyorhinis* and *mycoplasma hyosynoviae* infections under experimental conditions. PLoS One..

[13] Salogni C, Capucchio MT, Colombino E, Pozzi P, Pasquali P, Alborali GL (2022). Bacterial polyarthritis in post-weaning pigs in a high-density swine breeding area in Italy. J Vet Diagn Investig..

[14] Miniats O, Spinato M, Sanford S (1989). *Actinobacillus suis* septicemia in mature swine: two outbreaks resembling erysipelas. Can Vet J..

[15] Yaeger MJ (1996). An outbreak of *Actinobacillus Suis* septicemia in grow/finish pigs. J Vet Diagn Investig..

[16] MacInnes J, Gottschalk M, Lone A, Metcalf D, Ojha S, Rosendal T (2008). Prevalence of *Actinobacillus pleuropneumoniae*, *Actinobacillus suis*, *Haemophilus parasuis*, *Pasteurella multocida*, and *Streptococcus suis* in representative Ontario swine herds. Can J Vet Res..

[17] Hagedorn-Olsen T, Nielsen NC, Friis NF, Nielsen J (1999). Department of Clinical Studies, the Royal Veterinary and Agricultural University, Copenhagen, Denmark. J Veterinary Med Ser A..

[18] Nielsen EO, Nielsen NC, Friis NF (2001). *Mycoplasma hyosynoviae* arthritis in grower-finisher pigs. J Veterinary Med Ser A..

[19] Arruda PHE, Gauger P (2019). Optimizing sample selection, collection, and submission to optimize diagnostic value. Diseases of Swine.

[20] Derscheid RJ, Rahe MC, Burrough ER, Schwartz KJ, Arruda B (2021). Disease diagnostic coding to facilitate evidence-based medicine: current and future perspectives. J Vet Diagn Investig..

[21] Markey B, Leonard F, Archambault M, Cullinane A, Maguire D (2013). Clinical veterinary microbiology.

[22] Buchan BW, Ledeboer NA (2014). Emerging Technologies for the Clinical Microbiology Laboratory. Clin Microbiol Rev..

[23] Burrough E, Schwartz A, Gauger P, Harmon K, Krull A, Schwartz K (2018). Comparison of postmortem airway swabs and lung tissue for detection of common porcine respiratory pathogens by bacterial culture and polymerase chain reaction assays. J Swine Health Prod..

[24] Gomes Neto JC, Bower L, Erickson BZ, Wang C, Raymond M, Strait EL (2015). Quantitative real-time polymerase chain reaction for detecting *mycoplasma hyosynoviae* and *mycoplasma hyorhinis* in pen-based oral, tonsillar, and nasal fluids. J Vet Sci..

[25] Trevisan G, Schwartz KJ, Burrough ER, Arruda B, Derscheid RJ, Rahe MC (2021). Visualization and application of disease diagnosis codes for population health management using porcine diseases as a model. J Vet Diagn Investig..

[26] Trevisan G, Linhares LCM, Schwartz KJ, Burrough ER, Magalhães E de S, Crim B, et al. Data standardization implementation and applications within and among diagnostic laboratories: integrating and monitoring enteric coronaviruses. J Vet Diagn Investig. 2021 ;33(3):457–468. 10.1177/10406387211002163 PMC8120076 33739188

[27] Trevisan G, Linhares LCM, Crim B, Dubey P, Schwartz KJ, Burrough ER (2019). Macroepidemiological aspects of porcine reproductive and respiratory syndrome virus detection by major United States veterinary diagnostic laboratories over time, age group, and specimen. PLoS One..

[28] USDA. US State Rankings – 2021 Inventory [Internet]. 2022 [cited 2023 Jan 9]. Available from: https://app.usda-reports.penguinlabs.net/?crop=hogs_breeding&statistic=inventory_head&year=2021

[29] Rademacher C, Pudenz C, Schulz L (2019). Impact assessment of new US Food and Drug Administration regulations on antibiotic use: a post-enactment survey of swine practitioners. J Swine Health Prod..

[30] Lekagul A, Tangcharoensathien V, Yeung S (2019). Patterns of antibiotic use in global pig production: a systematic review. Vet Anim Sci..

[31] Clavijo MJ, Sreevatsan S, Johnson TJ, Rovira A (2019). Molecular epidemiology of *mycoplasma hyorhinis* porcine field isolates in the United States. PLoS One..

[32] Spiegel K, O’Hara K, Vanicek C, Beemer O. United States Department of Agriculture. 2022 [Cited 2023 Jan 9]. Swine Hemorrhagic Fevers: African and Classical Swine Fevers Integrated Surveillance Plan. Available from: https://www.aphis.usda.gov/animal_health/downloads/animal_diseases/swine/hemorrhagic-fevers-integrated-surveillance-plan.pdf.

[33] Estrada AA, Gottschalk M, Rossow S, Rendahl A, Gebhart C, Marthaler DG (2019). Serotype and genotype (multilocus sequence type) of *Streptococcus suis* isolates from the United States serve as predictors of Pathotype. J Clin Microbiol..

[34] Mugabi R, Silva APSP, Hu X, Gottschalk M, Aragon V, Macedo NR (2023). Molecular characterization of Glaesserella parasuis strains circulating in North American swine production systems. BMC Vet Res.

[35] Macedo N, Gottschalk M, Strutzberg-Minder K, Van CN, Zhang L, Zou G (2021). Molecular characterization of *Glaesserella parasuis* strains isolated from North America, Europe and Asia by serotyping PCR and LS-PCR. Vet Res..

[36] Kikuti M, Paploski IAD, Pamornchainavakul N, Picasso-Risso C, Schwartz M, Yeske P (2021). Emergence of a new lineage 1C variant of porcine reproductive and respiratory syndrome virus 2 in the United States. Front Vet Sci..

[37] Vötsch D, Willenborg M, Weldearegay YB, Valentin-Weigand P (2018). *Streptococcus suis* – the “two faces” of a Pathobiont in the porcine respiratory tract. Front Microbiol..

[38] Pan Z, Ma J, Dong W, Song W, Wang K, Lu C (2015). Novel variant serotype of *Streptococcus suis* isolated from piglets with meningitis. Appl Environ Microbiol..

[39] Pan Z, Ma Y, Ma J, Dong W, Yao H (2017). Acute meningitis of piglets and mice caused by co-infected with *Streptococcus suis* and *Aerococcus viridans*. Microb Pathog..

[40] Ni HB, Gong QL, Zhao Q, Li XY, Zhang XX (2020). Prevalence of *Haemophilus parasuis“Glaesserella parasuis*” in pigs in China: a systematic review and meta-analysis. Prev Vet Med..

[41] Palzer A, Haedke K, Heinritzi K, Zoels S, Ladinig A, Ritzmann M (2015). Associations among *Haemophilus parasuis*, *mycoplasma hyorhinis*, and porcine reproductive and respiratory syndrome virus infections in pigs with polyserositis. Can Vet J..

[42] Palzer A, Ritzmann M, Hafner-Marx A, Wolf G, Heinritzi K (2006). Detection of *Haemophilus parasuis* and *mycoplasma hyorhinis* in swine and association of those pathogens with clinical and pathological-anatomic findings. Dtsch Tierarztl Wochenschr..

[43] Lee JA, Oh YR, Hwang MA, Lee JB, Park SY, Song CS (2016). *Mycoplasma hyorhinis* is a potential pathogen of porcine respiratory disease complex that aggravates pneumonia caused by porcine reproductive and respiratory syndrome virus. Vet Immunol Immunopathol..

[44] Merodio M, Groeltz J, Piñeyro P, Derscheid R (2022). Retrospective analysis of *Mycoplasma hyorhinis* pulmonary and systemic infection in diagnostic cases with correlation of qPCR Ct values and detection by RNAscope®. In: 53rd Annual Meeting of the American Association of Swine Veterinarians.

[45] Clavijo MJ, Murray D, Oliveira S, Rovira A (2017). Infection dynamics of *mycoplasma hyorhinis* in three commercial pig populations. Vet Rec..

[46] Van Ostaaijen J, Frey J, Rosendal S, MacInnes JI (1997). *Actinobacillus suis* strains isolated from healthy and diseased swine are clonal and carry apxICABDvar. *Suis* and apxIICAvar. *Suis* toxin genes. J Clin Microbiol..

[47] Ojha S, Lacouture S, Gottschalk M, MacInnes JI (2010). Characterization of colonization-deficient mutants of *Actinobacillus suis*. Vet Microbiol..

[48] Mahan-Riggs E (2022). Three cases of Actinobacillus suis in eastern North Carolina. J Swine Health Prod..

[49] Roos LR, Surendran Nair M, Rendahl AK, Pieters M (2019). *Mycoplasma hyorhinis* and *mycoplasma hyosynoviae* dual detection patterns in dams and piglets. PLoS One..

[50] Pillman D, Surendran Nair M, Schwartz J, Pieters M (2019). Detection of *mycoplasma hyorhinis* and *mycoplasma hyosynoviae* in oral fluids and correlation with pig lameness scores. Vet Microbiol..

[51] Hallowell A, Pierdon M (2022). Effects of lameness on productivity and longevity for sows in pen gestation. J Swine Health Prod..

[52] Heinonen M, Peltoniemi O, Valros A (2013). Impact of lameness and claw lesions in sows on welfare, health and production. Livest Sci..

[53] Canning P, Costello N, Mahan-Riggs E, Schwartz K, Skoland K, Crim B (2019). Retrospective study of lameness cases in growing pigs associated with joint and leg submissions to a veterinary diagnostic laboratory. J Swine Health Prod..

[54] Brockmeier SL, Halbur PG, Thacker EL (2014). Porcine respiratory disease complex. Polymicrobial Diseases.

[55] Sarli G, D’Annunzio G, Gobbo F, Benazzi C, Ostanello F (2021). The role of pathology in the diagnosis of swine respiratory disease. Vet Sci..

[56] Siqueira FM, Pérez-Wohlfeil E, Carvalho FM, Trelles O, Schrank IS, Vasconcelos ATR (2017). Microbiome overview in swine lungs. PLoS One..

[57] Santos J, Schwartz K, Derscheid R, Magstadt D, Burrough E, Gauger P, et al. Distribution and characterization of Streptococcus suis strains of clinical importance within the US swine herd. 2022. 10.54846/am2022/110.

