# Supplementary material for: Detection and disease diagnosis trends (2017–2022) for Streptococcus suis, Glaesserella parasuis, Mycoplasma hyorhinis, Actinobacillus suis and Mycoplasma hyosynoviae at Iowa State University Veterinary Diagnostic Laboratory
Source: BMC Vet Res. 2023 Dec 12;19:268. doi: 10.1186/s12917-023-03807-w (PMC10714645; doi:10.1186/s12917-023-03807-w)
Supplement: Supplementary file 1 — Additional file 1. [file 12917_2023_3807_MOESM1_ESM.docx]

**Supplementary Tables**

**Supplementary T1.** Summary of terms used in this study.

| Term | Definition |
| --- | --- |
| Accession number | Unique numerical code linked to each case in the Iowa State University Veterinary Diagnostic Laboratory Information Management System (ISU-LIMS). |
| Agent detection | Isolation of the agent by bacterial culture and/or a positive PCR result. |
| Body system | Body systems selected for this study originated from the ISU VDL classification scheme (Derscheid et al., 2021). Cardiovascular comprised pathological findings related to the heart and pericardium, musculoskeletal to the joints, nervous to the central nervous system tissues, respiratory to the lungs, and systemic to the serosas and fibrinous exudates. |
| Case  (unit of analyses) | Materials provided by submitting veterinarian, including demographic information of farm (type of site, localization), characteristics of pig (age and clinical signs), and specimens and sample types to be used to detect pathogens or diagnose disease, as well as the test results and diagnosis associated with the case provided by the ISU VDL. Each case receives a unique accession number. |
| Disease diagnosis | Disease code based on the pathologic assessment, clinical history, and laboratory testing assigned by a diagnostician. The disease code was categorized by body system(s), insult(s), lesion(s), and etiology(ies). |
| Infectious etiology | Infectious agent detected with disease confirmation. |
| Insult | Insult is the type of pathological involvement in the case, such as viral, bacterial, parasitic, neoplastic, toxic, anomaly, metabolic, etc. |
| Lesion | A lesion would be a structural deviation from the normality in tissue(s). However, because a case can be comprised by more than one pig, this study used “lesion” to generalize to the case level the gross and microscopic pathology findings. Thus, this study includes terms appropriate for bacterial insult, such as arthritis (containing also synovitis), bronchopneumonia, endocarditis, meningitis (encephalitis), serositis (pleuritis, pericarditis, epicarditis, peritonitis, and polyserositis), and sepsis. |
| Specimen | Specific set of tissues and specimens utilized for the detection and diagnosis for the five bacterial agents. |

**Supplementary T2.** Diagnostic codes (Dx Codes) related to *S. suis*, *G. parasuis*, *M. hyorhinis*, *A. suis*, and *M. hyosynoviae* disease diagnoses. Detailed description of Dx Codes can be assessed in Derscheid et al. (21). The Dx Codes are simply listed in alphabetical order.

| Etiology Code | Lesions Code | Insult Code | Body System Code |
| --- | --- | --- | --- |
| *S. suis* | Arthritis | Bacterial | Cardiovascular-Blood-Endocrine-Immune |
|  | Bronchopneumonia |  | Musculoskeletal |
|  | Endocarditis |  | Nervous |
|  | Encephalitis |  | Respiratory |
|  | Epicarditis |  | Systemic |
|  | Meningitis |  |  |
|  | Pleuritis |  |  |
|  | Sepsis |  |  |
|  | Serositis |  |  |
| *G. parasuis* | Arthritis | Bacterial | Cardiovascular-Blood-Endocrine-Immune |
|  | Bronchopneumonia |  | Musculoskeletal |
|  | Encephalitis |  | Nervous |
|  | Epicarditis |  | Respiratory |
|  | Meningitis |  | Systemic |
|  | Pleuritis |  |  |
|  | Sepsis |  |  |
|  | Serositis |  |  |
| *M. hyorhinis* | Arthritis | Bacterial | Cardiovascular-Blood-Endocrine-Immune |
|  | Bronchopneumonia |  | Musculoskeletal |
|  | Epicarditis |  | Respiratory |
|  | Meningitis |  | Systemic |
|  | Pleuritis |  |  |
|  | Serositis |  |  |
| *A. suis* | Arthritis | Bacterial | Cardiovascular-Blood-Endocrine-Immune |
|  | Bronchopneumonia |  | Musculoskeletal |
|  | Endocarditis |  | Nervous |
|  | Encephalitis |  | Respiratory |
|  | Epicarditis |  | Systemic |
|  | Meningitis |  |  |
|  | Pleuritis |  |  |
|  | Sepsis |  |  |
|  | Serositis |  |  |
| *M. hyosynoviae* | Arthritis | Bacterial | Musculoskeletal |

**Supplementary T3.** List of diagnostic codes included as the denominator for evaluation of the diagnostic trend. The numerators are in bold.

| Combination Etiology_Lesion | | Etiology complete name | Denominators by Lesions |
| --- | --- | --- | --- |
| **AS_Arthritis** | **Actinobacillus_suis_** | **Arthritis** |  |
| Ecoli_Arthritis | E._coli_ | Arthritis |  |
| Eryrh_Arthritis | Erysipelothrix_rhusiopathiae_ | Arthritis |  |
| **GPS_Arthritis** | **Glaesserella_parasuis_** | **Arthritis** |  |
| **MHR_Arthritis** | **Mycoplasma_hyorhinis_** | **Arthritis** |  |
| **MHS_Arthritis** | **Mycoplasma_hyosynoviae_** | **Arthritis** |  |
| Mhsp_Arthritis | Mycoplasma_sp_ | Arthritis |  |
| Salm_Arthritis | Salmonella__ | Arthritis |  |
| **SSuis_Arthritis** | **Streptococcus_suis_** | **Arthritis** |  |
| Stapaur_Arthritis | Staphylococcus_aureus_ | Arthritis |  |
| Stasp_Arthritis | Staphylococcus_sp_ | Arthritis |  |
| Strepequi_Arthritis | Streptococcus_equi_subspecie-equi | Arthritis |  |
| Trupyo_Arthritis | Trueperella_pyogenes_ | Arthritis |  |
| Nonspeci_Arthritis | Non-specified bacterial insult | Arthritis |  |
| Ape_Bronchopneumonia | Actinobacillus_equuli_ | Bronchopneumonia |  |
| App_Bronchopneumonia | Actinobacillus_pleuropneumoniae_ | Bronchopneumonia |  |
| Apsp_Bronchopneumonia | Actinobacillus_sp_ | Bronchopneumonia |  |
| **AS_Bronchopneumonia** | **Actinobacillus_suis_** | **Bronchopneumonia** |  |
| Borbron_Bronchopneumonia | Bordetella_bronchiseptica_ | Bronchopneumonia |  |
| Ecoli_Bronchopneumonia | E._coli_ | Bronchopneumonia |  |
| **GPS_Bronchopneumonia** | **Glaesserella_parasuis_** | **Bronchopneumonia** |  |
| Kleb_Bronchopneumonia | Klebsiella_sp_ | Bronchopneumonia |  |
| Mhp_Bronchopneumonia | Mycoplasma_hyopneumoniae_ | Bronchopneumonia |  |
| **MHR_Bronchopneumonia** | **Mycoplasma_hyorhinis_** | **Bronchopneumonia** |  |
| Mycobacterium_Bronchopneumonia | Mycobacterium _sp_ | Bronchopneumonia |  |
| Pasmult_Bronchopneumonia | Pasteurella_multocida_ | Bronchopneumonia |  |
| **SSuis_Bronchopneumonia** | **Streptococcus_suis_** | **Bronchopneumonia** |  |
| Stasp_Bronchopneumonia | Staphylococcus_sp_ | Bronchopneumonia |  |
| Strepequi_Bronchopneumonia | Streptococcus_equi_subspecie-equi | Bronchopneumonia |  |
| Trupyo_Bronchopneumonia | Trueperella_pyogenes_ | Bronchopneumonia |  |
| Nonspeci_Bronchopneumonia | Non-specified bacterial insult | Bronchopneumonia |  |
| Ecoli_Endocarditis | E._coli_ | Endocarditis |  |
| Eryrh_Endocarditis | Erysipelothrix_rhusiopathiae_ | Endocarditis |  |
| **SSuis_Endocarditis** | **Streptococcus_suis_** | **Endocarditis** |  |
| Strepequi_Endocarditis | Streptococcus_equi_subspecie-equi | Endocarditis |  |
| Strepgalloly_Endocarditis | Streptococcus_gallolyticus_ | Endocarditis |  |
| Trupyo_Endocarditis | Trueperella_pyogenes_ | Endocarditis |  |
| Nonspeci_Endocarditis | Non-specified bacterial insult | Endocarditis |  |
| **AS_Meningitis** | **Actinobacillus_suis_** | **Meningitis** |  |
| Ecoli_Meningitis | E._coli_ | Meningitis |  |
| **GPS_Meningitis** | **Glaesserella_parasuis_** | **Meningitis** |  |
| Salm_Meningitis | Salmonella__ | Meningitis |  |
| **SSuis_Meningitis** | **Streptococcus_suis_** | **Meningitis** |  |
| Trupyo_Meningitis | Trueperella_pyogenes_ | Meningitis |  |
| Nonspeci_Meningitis | Non-specified bacterial insult | Meningitis |  |
| Ape_Sepsis | Actinobacillus_equuli_ | Sepsis |  |
| App_Sepsis | Actinobacillus_pleuropneumoniae_ | Sepsis |  |
| **AS_Sepsis** | **Actinobacillus_suis_** | **Sepsis** |  |
| Ecoli_Sepsis | E._coli_ | Sepsis |  |
| Eryrh_Sepsis | Erysipelothrix_rhusiopathiae_ | Sepsis |  |
| **GPS_Sepsis** | **Glaesserella_parasuis_** | **Sepsis** |  |
| Kleb_Sepsis | Klebsiella_sp_ | Sepsis |  |
| **MHR_Sepsis** | **Mycoplasma_hyorhinis_** | **Sepsis** |  |
| Pasmult_Sepsis | Pasteurella_multocida_ | Sepsis |  |
| Salm_Sepsis | Salmonella__ | Sepsis |  |
| **SSuis_Sepsis** | **Streptococcus_suis_** | **Sepsis** |  |
| Stasp_Sepsis | Staphylococcus_sp_ | Sepsis |  |
| Strepequi_Sepsis | Streptococcus_equi_subspecie-equi | Sepsis |  |
| Strepzoo_Sepsis | Streptococcus_equi_subspecie-zooepidemicus | Sepsis |  |
| Trupyo_Sepsis | Trueperella_pyogenes_ | Sepsis |  |
| Nonspeci_Sepsis | Non-specified bacterial insult | Sepsis |  |
| **AS_Serositis** | **Actinobacillus_suis_** | **Serositis** |  |
| Ecoli_Serositis | E._coli_ | Serositis |  |
| **GPS_Serositis** | **Glaesserella_parasuis_** | **Serositis** |  |
| Mhbo_Serositis | Mycoplasma_bovis_ | Serositis |  |
| **MHR_Serositis** | **Mycoplasma_hyorhinis_** | **Serositis** |  |
| Pasmult_Serositis | Pasteurella_multocida_ | Serositis |  |
| **SSuis_Serositis** | **Streptococcus_suis_** | **Serositis** |  |
| Strepequi_Serositis | Streptococcus_equi_subspecie-equi | Serositis |  |
| Trupyo_Serositis | Trueperella_pyogenes_ | Serositis |  |
| Nonspeci_Serositis | Non-specified bacterial insult | Serositis |  |
